# Phytochemicals from a Desert Crop, Sand Rice (Agriophyllum squarrosum), and Their Inflammatory Activity

**DOI:** 10.1021/acsomega.5c01825

**Published:** 2025-05-21

**Authors:** Ping Hai, Qiang Li, Xiao Fei Ma, Hai Yan Jia, Yun Qing He, Jin Yang, Xian Yan Li, Zhi Qiang Luo, Mei Ling Yang, Yuan Gao, Hong Peng Wang, Jian Yang

**Affiliations:** † Key Lab of Process Analysis and Control of Sichuan Universities, Faculty of Materials and Chemical Engineering, 91614 Yibin University, Yibin 644000, China; ∥ State Key Laboratory for Quality Ensurance and Sustainable Use of Dao-di Herbs, National Resource Center for Chinese Materia Medica, China Academy of Chinese Medical Sciences, Beijing 100700, China; § 71046 Evaluation and Research Center of Daodi Herbs of Jiangxi Province, Ganjiang New District, Nanchang 330000, China; ‡ Key Laboratory of Stress Physiology and Ecology in Cold and Arid Regions of Gansu Province, Department of Ecology and Agriculture Research, Northwest Institute of Eco-Environment and Resources, Chinese Academy of Sciences, Lanzhou 730000, China; ⊥ State Key Laboratory Breeding Base of Green Chemistry-Synthesis Technology, College of Chemical Engineering, Zhejiang University of Science and Technology, Hangzhou 310032, China

## Abstract

Agriophyllum squarrosum (sand rice), a resilient desert plant with ecological and nutritional significance, has applications in food, forage, and traditional medicine. Despite its traditional use in China for treating inflammatory symptoms such as ophthalmia, urethritis, and oral ulcers, limited phytochemical studies restrict its pharmacological exploration. In this study, ten undescribed isoflavanone derivatives, including dihydroisoflavanones (**1**–**6**) and coumaronochromones (**7**–**10**), along with thirty-six known compounds (**11**–**46**), were isolated from A. squarrosum. The new structures were determined by NMR, HRESIMS, and DFT calculations of their NMR and ECD data, which also led to the structural revision of suaeglaucin C. Anti-inflammatory assays revealed compounds **5** and **21** as potent inhibitors, outperforming L-NMMA, and the structure–activity relationship of the optically active dihydroisoflavones was briefly discussed. All compounds were isolated from this plant for the first time, except for compounds **11**, **19**, and **20**.

## Graphical abstract

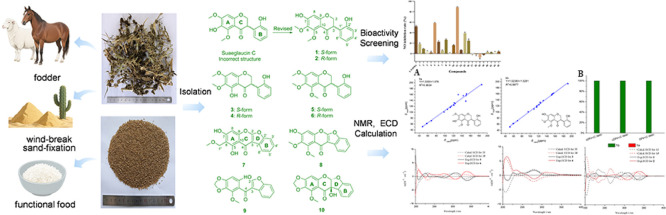

## 1 Introduction

Agriophyllum squarrosum (L.) Moq. (sand rice), an annual pioneer desert plant of the Chenopodiaceae family mainly distributed in the mobile sand and semifixes dunes of Central Asian, the Caucasus, Mongolia, and Siberia,^1,2^ is an ideal crop with outstanding ecological characteristics in arid and semiarid regions. This plant can tolerate extremely high temperatures, and its withered plant body can reduce wind velocity by at least 90% and fix sand, playing a critical role in fragile desert ecosystems.^3^ It is also capable of surviving in alpine regions such as the Qinghai-Tibet Plateau,^2^ and is thus a rich source of carbon and nitrogen in harsh ecological environments. Although it grows in infertile soils, A. squarrosum has high biomass and a high concentration of nutrients in its edible grains, which provides rich and balanced nutrition comparable to Chenopodium quinoa (also belonging to the family Chenopodiaceae), a healthy and nutritious crop recommended by the United Nations Food and Agriculture Organization.^4^ The seed of A. squarrosum is also known as a good plant-based food that can be used to develop various functional foods and beverages or be mixed with other grains in different recipes.^5,6^ The leaves and stems are suitable for ruminants as a feed resource. Recently, Liang et al. reported that feeding lambs with the whole plants of A. squarrosum can improve meat quality by increasing water-holding capacity, reducing muscle fiber diameter, and increasing the density of the meat without compromising growth.^7^ Their findings also provide strong evidence supporting the benefits of A. squarrosum as a dietary supplement for ruminants, particularly in reducing blood lipids and enhancing immune response and anti-inflammatory capacity.^6^

In addition to its value for food, forage, and ecology,^1,8–10^ A. squarrosum was also used as a folk medicine in China to treat inflammatory symptoms such as ophthalmia, jaundice, urethritis, oral ulcers, and headaches.^11^ The aqueous extracts of A. squarrosum have shown diverse activities,^6,9,12^ including antidiabetic, antioxidant, and antihyperlipidemic properties. The only phytochemical study on A. squarrosum was conducted by Li et al., which revealed the presence of alkaloids,^13^ coumarins,^11^ flavonoids,^14^ isoflavonoids,^13^ and triterpenoid saponins.^15^

In this study, ten undescribed isoflavone derivatives (**1**–**10**) and thirty-six known compounds (**11**–**46**) were isolated and elucidated (Figures 1 and 2) from this plant. Three pairs of enantiomers, (+)-**1**, (−)-**2**, (+)-**3**, (−)-**4**, (+)-**5**, and (−)-**6**, were separated by chiral-phase HPLC resolution, and their absolute configurations were elucidated by spectroscopic analyses and quantum chemical electronic circular dichroism (ECD) calculations. A previously reported compound, suaeglaucin C, displayed inconsistencies between the assigned NMR data and the established structure;^16^ thus, a revised structure was presented (Figure 1). The anti-inflammatory capabilities of the isolated compounds were assessed to explore their therapeutic potential. The structure–activity relationship (SAR) of the dihydroisoflavone derivatives is also briefly discussed. Herein, we report the details of isolation, structural elucidation, anti-inflammatory activity, and SAR of the phytochemicals from A. squarrosum.

**Figure 1:**
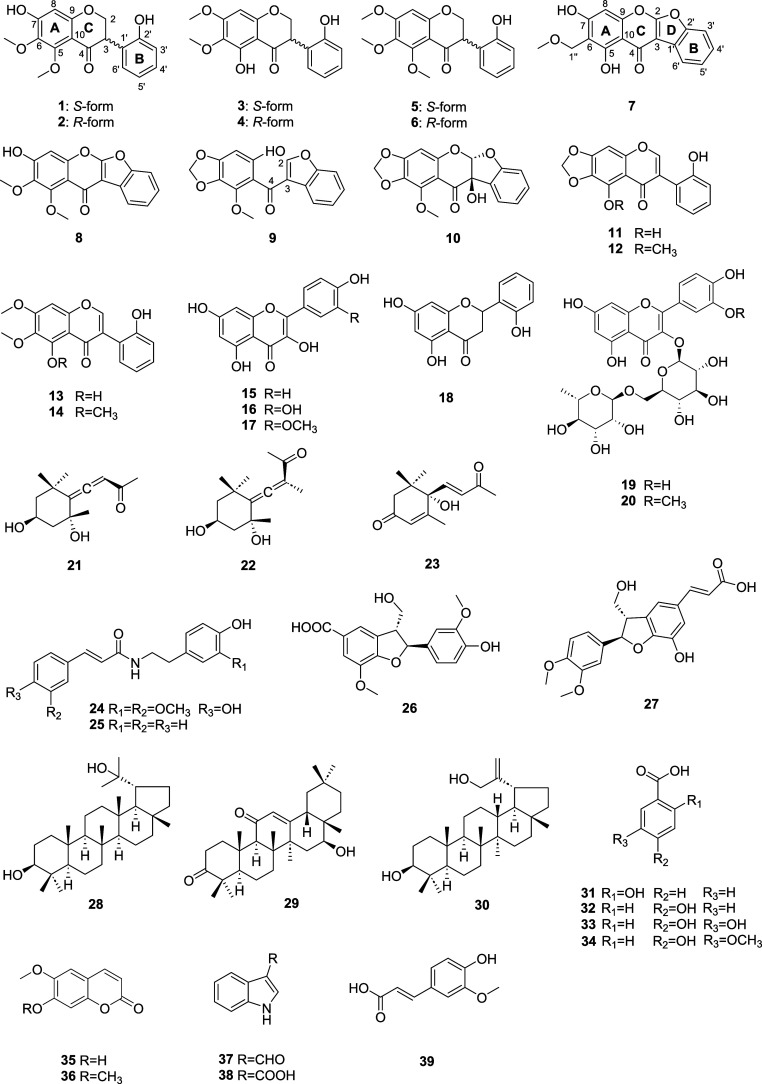
Structures of **1**–**39** from the aerial parts of A. squarrosum.

**Figure 2:**
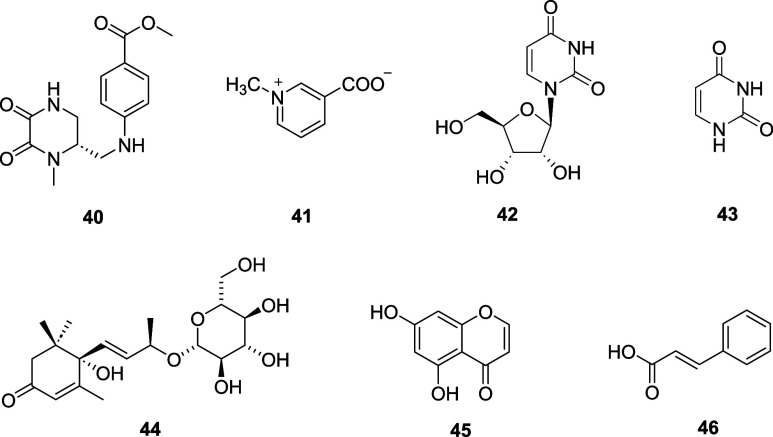
Structures of **40**–**46** from the seeds of A. squarrosum.

## 2 Materials and Methods

### 2.1 General Experimental Procedures

UV spectra were obtained by using a Shimadzu UV2401PC spectrophotometer. ESIMS and HRESIMS were performed on an Agilent G6230 time-of-flight mass spectrometer. IR spectra were obtained on a Thermo Nicolet iS10 spectrometer with KBr pellets. NMR spectra were acquired with a Bruker Avance III 500 or 600 instrument at room temperature. Silica gel (200–300 mesh, Qingdao Marine Chemical Factory, China) and Sephadex LH-20 (Amersham Biosciences, Sweden) were used for column chromatography. Semipreparative HPLC was performed on an AS20005 series (Hanbon, China) using a 5C_18_-AR-II column (5 μm, 10 × 250 mm, 3.0 mL/min, Nacalai Tesque, Japan) or a Chiralpak IG chiral column (5 μm, 250 mm × 10 mm, 3.0 mL/min, Daicel Chiral Technologies Co. Ltd., Tokyo, Japan), and a P3500 series (Dalian Elite, China) using an XAmid column (10 μm, 20 × 250 mm, 15 mL/min, Dalian Elite, China).

### 2.2 Plant Material

The aerial parts and seeds of A. squarrosum were purchased from a commercial supplier (Sunshine Fufan Breeding Professional Cooperative, Gulang, Gansu, China). The voucher specimens were identified by Prof. Xiaofei Ma (Department of Ecology and Agricultural Research, Northwest Institute of Eco-Environment and Resources, Chinese Academy of Sciences) and deposited at the Laboratory of Phytochemistry, Yibin University.

### 2.3 Extraction and Isolation

The aerial parts of A. squarrosum (20 kg) were cut into small segments, which were extracted with methanol (each 40 L, 48 h) at room temperature three times. The resulting methanol extracts (ca. 1.7 kg) were subjected to silica gel column chromatography (CC), using petroleum ether (PE)/EtOAc (100:0 to 0:100 gradient), and then EtOAc/MeOH (90:10 to 0:100), to give fractions A–Y. Fr. I was separated on silica gel, and the CC was eluted with PE/EtOAc (98:2 → 1:1) to provide subfractions (I1–I9). Fr. I5 was subjected to Sephadex LH-20 CC (CH_2_Cl_2_/MeOH, 1:1), silica gel CC (CH_2_Cl_2_/MeOH = 100:1), and thin-layer chromatography to obtain compounds **3**/**4** (11.1 mg), **7** (10.3 mg), **10** (0.8 mg), and **29** (8.2 mg). The racemic mixture **3**/**4** was further resolved by semipreparative chiral HPLC eluting with *n*-hexane/EtOH (85/15, V/V, 3.0 mL/min) to give **3** (2.4 mg) and **4** (2.0 mg). Fr. J was separated on MCI gel CC (30% → 100% MeOH) to obtain 6 fractions (J1–J6). **8** (16.9 mg), **11** (23.4 mg), **12** (15.6 mg), **13** (10.8 mg), **14** (32.0 mg), **35** (10.1 mg), **36** (4.4 mg), and **37** (3.0 mg) were obtained from Fr. J5 by repeated silica gel CC (PE/EtOAc 100:1 → 0:100) and Sephadex LH-20 (CH_2_Cl_2_/MeOH = 1:1). Fraction K was separated by silica gel CC eluting with a gradient system of CH_2_Cl_2_/MeOH (99:1 → 80:20) to obtain eight fractions (K1–K8). The enantiomeric pair **1**/**2** was obtained from Fr. K2 by Sephadex LH-20 CC (CH_2_Cl_2_/MeOH = 1:1) and semipreparative HPLC using a 5C_18_-AR-II column (75% MeOH), and then resolved into **1** (10.4 mg) and **2** (8.1 mg) by semipreparative chiral HPLC using a CHIRALCEL IG chiral column (*n*-hexane/EtOH = 85/15, V/V, 3.0 mL/min). Fr. K5 was separated by Sephadex LH-20 (CH_2_Cl_2_/MeOH = 1:1) and silica gel CC (PE/EtOAc, 90:10→0:100) to obtain compounds **9** (5.1 mg), **28** (20.1 mg), and **30** (6.8 mg). Compounds **5** and **6** were obtained as a racemic mixture from Fr. K6 by Sephadex LH-20 CC (CH_2_Cl_2_/MeOH, 1:1) and semipreparative HPLC using a 5C_18_-AR-II column (80% MeOH), and then resolved into **5** (18.3 mg) and **6** (14.7 mg) by semipreparative HPLC using a CHIRALCEL IG chiral column with *n*-hexane/EtOH (84/16, V/V, 3.0 mL/min). Fraction P was separated using silica gel CC and eluted with CH_2_Cl_2_/MeOH (30:1→0:100) to obtain 6 fractions (P1–P6). Compounds **15** (33.9 mg), **16** (23.7 mg), **17** (12.2 mg), **18** (9.7 mg), **21** (8.8 mg), **22** (5.7 mg), **23** (11.4 mg), **24** (40.6 mg), and **25** (2.2 mg) were obtained from Fr. P4 by Sephadex LH-20 (CH_2_Cl_2_/MeOH, 1:1), silica gel CC (CH_2_Cl_2_/MeOH, 35:1 → 0:100), and then semipreparative HPLC (60% MeOH). Compounds **26** (3.5 mg), **27** (2.7 mg), **31** (7.5 mg), **32** (5.0 mg), **33** (12.4 mg), **34** (32.1 mg), **38** (13.7 mg), and **39** (14.3 mg) were obtained from Fr. U by Sephadex LH-20 CC (CH_2_Cl_2_/MeOH, 1:1), silica gel CC (CH_2_Cl_2_/MeOH, 25:1 → 0:100), and then semipreparative HPLC (65% MeOH). Fraction W was separated by silica gel column eluting with a gradient system of CH_2_Cl_2_/MeOH (15:1 → 0:100) to obtain **19** (73.1 mg) and **20** (44.2 mg).

The seeds of A. squarrosum (30 kg) were extracted three times with methanol (each 40 L, 48 h) at room temperature. The extract (ca. 1.5 kg) was separated by CC over silica gel and eluted with petroleum ether PE/EtOAc (100:0 → 0:100 gradient) to give nine fractions A-I. Fr. C (9.0 g) was separated by Sephadex LH-20 CC (CH_2_Cl_2_/MeOH, 1:1) to yield fractions C1–C3. Fr. C3 was further separated on semipreparative HPLC with 85% MeOH to yield compound **40** (10.3 mg). Fr. D (15.6 g) was subjected to silica gel CC eluted with EtOAc/MeOH (100:0 → 0:100 gradient), Sephadex LH-20 CC (CH_2_Cl_2_/MeOH, 1:1), and semipreparative HPLC with 75% MeOH to yield compounds **45** (2.0 mg) and **46** (3.1 mg). Compounds **42** (13.4 mg), **43** (9.7 mg), and **44** (17.5 mg) were obtained from Fr. G by Sephadex LH-20 CC (CH_2_Cl_2_/MeOH, 1:1), silica gel CC (EtOAc/MeOH = 50:1→0:100), and semipreparative HPLC (MeOH/H_2_O, 15:85). Fr. H (742.5 mg) was separated by semipreparative HPLC (XAmid column) eluting with 5% MeOH and further subjected to Sephadex LH-20 CC (H_2_O/MeOH, 1:1) to give compound **41** (20.2 mg).

(±)-Agrisquarin A (**1**/**2**): yellowish oil; IR (KBr) ν_max_ 3402, 2943, 2836, 1610, 1483, 1457, 1281, 1161, 1088, and 1026 cm^–1^; ESIMS: *m*/*z* 315 [M – H]^−^; HRESIMS: *m*/*z* 315.0875 (calcd for C_17_H_15_O_6_, 315.0874); ^1^H and ^13^C NMR data, see Table 1.

**Table 1:** NMR Spectroscopic Data for **1**–**6** (*δ* in ppm, *J* in Hz)

	1/2^a^		1/2^b^	Suaeglaucin C^b^		3/4^b^		5/6^b^	
No.	δ_H_	δ_C_	δ_H_	δ_H_	δ_C_	δ_H_	δ_C_	δ_H_	δ_C_
2	4.59, t, 10.9	71.1, CH_2_	4.75, dd, 11.9, 4.5	4.73, dd, 11.9, 4.5	69.2, CH_2_	4.87, dd, 11.8, 5.2	69.7, CH_2_	4.92, dd, 11.9, 3.4	69.5, CH_2_
	4.37, dd,10.8, 5.4		4.90, dd, 11.9, 3.4	4.89, dd, 11.9, 3.5		4.71, dd, 11.8, 4.9		4.76, 11.9, 4.6	
3	4.07, dd,10.8, 5.4	51.0, CH	3.97, dd, 4.5, 3.4	3.97, dd, 4.5, 3.5	46.9, CH	4.12, t, 5.0	45.6, CH	3.98, t, 4.0	46.9, CH
4		193.3, C			191.9, C		197.3, C		191.9, C
5		156.1, C			135.5, C		155.6, C		155.1, C
6		137.7, C			157.0, C		130.7, C		137.8, C
7		159.5, C			155.5, C		161.8, C		160.8, C
8	6.22, s	100.5, CH	6.37, s	6.36, s	98.9, CH	6.08, s	91.6, CH	6.30, s	96.0, CH
9		162.0, C			160.2, C		158.9, C		160.2, C
10		110.1, C			107.3, C		102.1, C		107.4, C
1’		124.0, C			123.3, C		122.3, C		123.5, C
2’		156.6, C			155.5, C		154.8, C		155.7, C
3′	6.79, dd, 7.7, 1.1	116.3, CH	6.97, d, 7.6	6.96, d, 7.9	118.0, CH	6.94, d, 7.6	117.6, CH	6.97, dd, 8.1, 1.2	118.2, CH
4’	7.10, td, 7.7, 1.6	129.7, CH	7.20, t, 7.7	7.18, dd, 7.9, 7.6	129.3, CH	6.93, m^c^	129.6, CH	7.19, td, 7.8, 1.6	129.4, CH
5′	6.77, td, 7.7, 1.1	120.6, CH	6.90, t, 7.6	6.89, dd, 7.6, 7.5	120.9, CH	7.22, td, 8.0, 1.6	121.4, CH	6.90, td, 7.6, 1.3	120.9, CH
6’	7.04, dd, 7.7, 1.6	131.7, CH	7.51, d, 7.8	7.49, d, 7.5	126.9, CH	7.42, brd, 7.4	127.9, CH	7.50, dd, 7.8, 1.4	126.8, CH
3-OH									
5-OMe/OH	3.86, s	61.9, CH_3_	3.89, s	3.88, s	61.3, CH_3_	11.59, s		3.90, s	61.5, CH_3_
6-OMe/OH	3.79, s	61.7, CH_3_	3.89, s	6.41, br.s	61.5, CH_3_	3.80, s	60.9, CH_3_	3.78, s	61.3, CH_3_
7-OMe/OH			6.44, br.s	3.88, s		3.91, s	56.3, CH_3_	3.90, s	56.3, CH_3_
2’-OH			8.45, br.s	8.40, br.s		7.27, brs		8.50, s	

a Measured at 600 MHz in CD_3_OD.

b Measured at 500 MHz in CDCl_3_.

c Overlapping signals.

(3*S*)-Agrisquarin A (**1**): yellowish oil; [α]_D_^20^ = +75.4 (*c* 0.10, MeOH); UV (MeOH) λ_max_ (log ε): 217 (4.5), 281 (4.7), and 321 (3.8) nm.

(3*R*)-Agrisquarin A (**2**): yellowish oil; [α]_D_^20^ = −86.4 (*c* 0.10, MeOH); UV (MeOH) λ_max_ (log ε): 218 (4.6), 281 (4.3), and 323 (3.8) nm.

(±)-Agrisquarin B (**3**/**4**): yellowish oil; IR (KBr) ν_max_ 3428, 2938, 1640, 1574, 1503, 1452, 1202, 1114, and 754 cm^–1^; ESIMS: *m*/*z* 339 [M + Na]^+^; HRESIMS: *m*/*z* 339.0836 (calcd for C_17_H_16_O_6_Na, 339.0839); ^1^H and ^13^C NMR data, see Table 1.

(3*S*)-Agrisquarin B (**3**): yellowish oil; [α]_D_^20^ = +29.2 (*c* 0.10, MeOH); UV (MeOH) λ_max_ (log ε): 232 (4.2), 287 (4.3), and 336 (3.4) nm.

(3*R*)-Agrisquarin B (**4**): yellowish oil; [α]_D_^20^ = −32.2 (*c* 0.10, MeOH); UV (MeOH) λ_max_ (log ε): 232 (4.5), 287 (4.5), and 337 (3.7) nm.

(±)-Agrisquarin C (**5**/**6**): yellowish oil; IR (KBr) ν_max_ 3392, 2940, 1602, 1487, 1455, 1271, 1204, 1106, 1019, and 824 cm^–1^; ESIMS: *m*/*z* 353 [M + Na]^+^; HRESIMS: *m*/*z* 2353.0993 (calcd for C_18_H_18_O_6_Na, 353.0996); ^1^H and ^13^C NMR data, see Table 1.

(3*S*)-Agrisquarin C (**5**): yellowish oil; [α]_D_^20^ = +45.0 (*c* 0.10, MeOH); UV (MeOH) λ_max_ (log ε): 215 (4.5), 277 (4.3), and 320 (3.7) nm.

(3*R*)-Agrisquarin C (**6**): yellowish oil; [α]_D_^20^ = −52.2 (*c* 0.10, MeOH); UV (MeOH) λ_max_ (log ε): 215 (4.5), 277 (4.2), and 320 (3.6) nm.

Agrisquarin D (**7**): yellow oil; UV (MeOH) λ_max_ (log ε): 201 (4.7), 251 (4.6), and 323 (4.2) nm; IR (KBr) ν_max_ 3239, 3076, 2923, 1636, 1454, 1171, 1077, and 745 cm^–1^; ESIMS: *m*/*z* 311 [M – H]; HRESIMS: *m*/*z* 311.0562 (calcd for C_17_H_11_O_6_, 311.0561); ^1^H and ^13^C NMR data, see Table 2.

**Table 2:** NMR Spectroscopic Data for **7**–**10** (*δ* in ppm, *J* in Hz)

	7^a^		8^b^		9^a^		10^a^	
No.	δ_H_	δ_C_	δ_H_	δ_C_	δ_H_	δ_C_	δ_H_	δ_C_
2		165.2, C		163.5, C	8.09, s	150.4, CH	6.28, s	109.3, CH
3		97.8, C		98.0, C		123.6, C		82.1, C
4		179.2, C		172.2, C		190.5, C		187.9, C
5		159.4, C		153.4, C		142.2, C		142.3, C
6		106.0, C		140.0, C	6.29, s	129.6,C		132.4, C
7		162.8, C		151.0, C		154.7, C		155.2, C
8	6.56, s	95.8, CH	6.94, s	100.5, CH		93.3, CH	6.29, s	94.5, CH
9		154.3, C		155.7, C		160.4, C		156.2, C
10		103.4, C		110.9, C		108.7, C		107.4, C
1’		122.5, C		123.1, C		125.1, CH		126.9, C
2’		149.3, C		148.6, C		154.9, C		160.4, C
3′	7.54, dd, 7.5, 1.2	111.3, CH	7.71, dd, 7.2, 1.2	111.5, CH	7.54, dd, 7.3, 1.4	111.6, CH	6.90, d, 8.1	110.6, CH
4’	7.39, td, 7.5, 1.4	125.4, CH	7.41, td, 7.2, 1.4	125.1, CH	7.36, td, 7.3, 1.4	125.1, CH	7.24, t, 8.0, 8.1	131.2, CH
5′	7.43, td, 7.5, 1.2	125.4, CH	7.44, td, 7.2, 1.2	125.2, CH	7.34, td, 7.3, 1.4	124.0, CH	6.94, t, 8.0, 8.1	122.4, CH
6’	8.06, dd, 7.5, 1.4	121.6, CH	8.00, dd, 7.2, 1.4	120.7, CH	7.89, dd, 7.3, 1.4	121.8, CH	7.22, d, 8.1	124.1, CH
1’’	4.88, s	68.0, CH_2_						
3-OH							4.63, s	
5-OMe/OH	13.26, s		3.79, s	60.9, CH_2_	11.62, s		4.00, s	60.3, CH_3_
6-OMe/OH	3.54, s	59.0, CH_3_	3.85, s	62.0, CH_2_				
7-OMe/OH	9.11, s		10.91, brs					
9-OMe					3.67, s	59.9, CH_3_		
–OCH_2_O–					5.96, s	101.4, CH_2_	5.91, s	101.9, CH_2_
							5.93, s	

a Measured at 600 MHz in CDCl_3_.

b Measured at 500 MHz in DMSO-*d*_6_.

Agrisquarin E (**8**): yellow oil; UV (MeOH) λ_max_ (log ε): 197 (4.7), 248 (4.5), and 310 (4.1) nm; IR (KBr) ν_max_ 3231, 3215, 2940, 1284, 1068, and 743 cm^–^;^1^ ESIMS: *m*/*z* 335 [M + Na]^+^; HRESIMS: *m*/*z* 335.0524 (calcd for C_17_H_12_O_6_Na, 335.0526); ^1^H and ^13^C NMR data, see Table 2.

Agrisquarin F (**9**): yellow oil; UV (MeOH) λ_max_ (log ε): 203 (4.8), 265 (sh), and 269 (4.0) nm; IR (KBr) ν_max_ 3410, 3134, 2911, 1620, 1553, 1094, 842, and 763 cm^–1^; ESIMS: *m*/*z* 335 [M + Na]^+^; HRESIMS: *m*/*z* 335.0524 (calcd for C_17_H_12_O_6_Na, 335.0526); ^1^H and ^13^C NMR data, see Table 2.

(±)-Agrisquarin G (**10**): yellowish oil; UV (MeOH) λ_max_ (log ε): 243 (3.3), 286 (3.2), and 336 (2.7) nm; IR (KBr) ν_max_ 3432, 2918, 2850, 1680, 1623, 1477, 1269, and 975 cm^–1^; ESIMS: *m*/*z* 351 [M + Na]^+^; HRESIMS: *m*/*z* 351.0473 (calcd for C_17_H_12_O_7_Na, 351.0475); ^1^H and ^13^C NMR data, see Table 2.

### 2.4 Anti-Inflammatory Activity Assay

The mouse macrophage cell line RAW264.7 was purchased from the Shanghai Institute of Biochemistry and Cell Biology and incubated in Dulbecco’s Modified Eagle Medium (Biological Industries, BioInd, Israel) supplemented with 10% fetal bovine serum (Biological Industries, BioInd, Israel) at 37 °C in a 5% CO_2_ incubator. The RAW264.7 cells were seeded in 96-well plates for 24 h. Then, they were treated with different concentrations (final concentration 50 μM) of compounds in the presence of LPS (1 μg/mL). After the cells were cultured overnight, the medium was used to detect NO production, and the absorbance at 570 nm was measured. MTS was added to the remaining medium to detect the cell survival rate and exclude the toxic effects of the compounds. The formula to calculate the inhibition rate is as follows: NO production inhibition rate (%) = (nondrug treatment group OD_570_ nm – sample group OD_570_ nm)/nondrug treatment group OD_570_ nm × 100%. IC_50_ was calculated using the Reed and Muench method.

## 3 Results and Discussion

### 3.1 Identification of Compounds 1–46

Compound **1**/**2** gave a molecular formula of C_17_H_16_O_6_ as evidenced by HRESIMS pseudo-molecular ion [M – H]^−^ at *m*/*z* 315.0875 (calcd 315.0874) and NMR data, requiring 10 degrees of unsaturation. Its IR spectrum suggested the presence of hydroxy (3202 cm^–1^) and conjugated carbonyl (1610 cm^– 1^) groups in **1**/**2**. The ^1^H NMR data (Table 1) of **1**/**2** revealed the presence of an ortho-disubstituted benzene ring [δ_H_ 6.79 (1H, dd, *J* = 7.7, 1.1 Hz), 7.10 (1H, td, *J* = 7.7, 1.6 Hz), 6.77 (1H, td, *J* = 7.7, 1.1 Hz), and 7.04 (1H, dd, *J* = 7.7, 1.6 Hz)], an aromatic proton (δ_H_ 6.22, 1H, s), a methine [δ_H_ 4.07 (1H, dd, *J* = 10.9, 5.4 Hz)] coupled to an oxygenated methylene [δ_H_ 4.59 (1H, t, *J* = 10.9, 10.8 Hz), δ_H_ 4.37 (1H, dd, *J* = 10.8, 5.4 Hz)], and two methoxyls [δ_H_ 3.86 (3H, s) and δ_H_ 3.79 (3H, s)]. The ^13^C NMR and DEPT spectra showed 17 carbon signals, including one carbonyl (δ_C_ 193.3), seven quaternary carbons (δ_C_ 162.0, 159.5, 156.1, 156.6, 137.7, 124.0, and 110.1), six methines (δ_C_ 131.7, 129.7, 120.6, 116.3, 100.5, and 51.0), a methylene (δ_C_ 71.1), and two methoxyls (δ_C_ 61.7 and 61.9). By analyzing these spectra, compound **1**/**2** was defined as an isoflavanone with two methoxy groups. The 1D NMR data of **1**/**2** in CDCl_3_ correspond well to those of suaeglaucin C,^16^ for which we found the structure reported was erroneously determined in terms of the position of OMe in ring A, as shown in Figure 3. The key signal (measured in CDCl_3_) reported for δ_C_ 135.5 was incorrectly designated to C-5, which eventually led **1**/**2** to structure suaeglaucin C, as an HMBC correlation from OMe to δ_C_ 135.5 was observable. Considering the shielding effect caused by the 5,7,9-trioxy substitution, this obvious upfield-shifted signal at δ_C_ 135.5 should be designated as C-6, not C-5. In our HMBC spectrum measured in CD_3_OD, the significant correlation from δ_H_ 3.79 (3H, s, OCH_3_) to δ_C_ 137.7 positioned this methoxy group at C-6. In addition, chemical shifts of Ar-OMe at about 60 ppm should be accompanied by *o*-substitutions on both sides of the OMe; otherwise, Ar-OMe signals appear around 56 ppm.^17^ According to this regulation, the two methoxy groups (δ_H_ 3.86/δ_C_ 61.9 and δ_H_ 3.79/δ_C_ 61.7) in **1**/**2** should be linked at C-5 and C-6, and the hydroxy group must be connected to C-7 (Figure 3). To further confirm the revised position of OMe, quantum computational methods were applied. The ^13^C NMR chemical shifts of **1/2** (**1a**) and suaeglaucin C (**1b**) were computed using the gauge-independent atomic orbitals (GIAO) method at the PCM-MPW1PW91/6–31G­(d, p) level based on the optimized conformers.^18^ The calculated ^13^C NMR spectra of compound **1/2** and suaeglaucin C showed that the chemical shifts of C-6 were both 131.7 ppm (Figure S2), which significantly deviated from the reported chemical shift of 157.0 ppm (C-6) for suaeglaucin C. This indicates a clear misassignment of the C-6 chemical shift in the previous literature. Further, the correlation coefficients (*R* ^2^ between the calculated and experimental data from linear regression analysis were 0.9977 for **1/2** and 0.9961 for suaeglaucin C, indicating that **1/2** was the favorable structure (Figure 4). The DP4+ probability analysis was applied to support the above deduction.^19^ As shown in Figure 5, the unscaled sDP4+, uDP4+, and DP4+ data of the carbons gave 100.00% confidence for **1/2**.^20^ The planar structure of suaeglaucin C is therefore revised to **1/2**. Compound **1/2** exhibited a specific rotation approaching zero and showed no Cotton effects in its ECD spectrum, suggesting a racemic mixture. Subsequent chiral resolution of **1/2** was performed by chiral prep-HPLC (Figure S1) to afford the anticipated enantiomers (+)-**1** and (−)-**2**, which showed opposite specific rotations and mirror-image-like ECD curves. The absolute configurations of **1** (3*R*) and **2** (3*S*) were determined by quantum chemical calculations of their theoretical ECD spectra (Figure 5). Finally, the structures of **1** and **2** were identified and named (*R*)-agrisquarin A and (*S*)-agrisquarin A, respectively.

**Figure 3:**
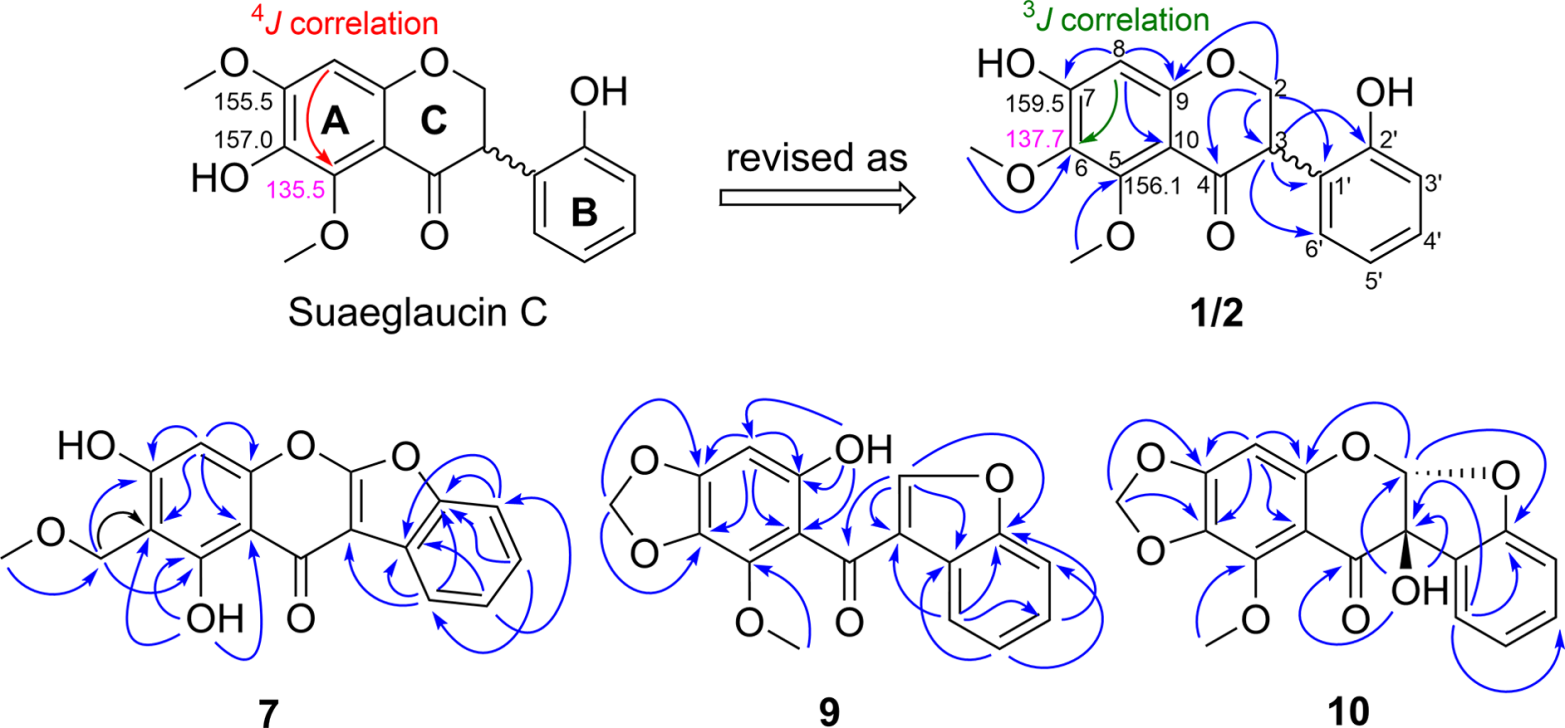
Key HMBC correlations of **1**/**2**, **7**, **9**, and **10**.

**Figure 4:**
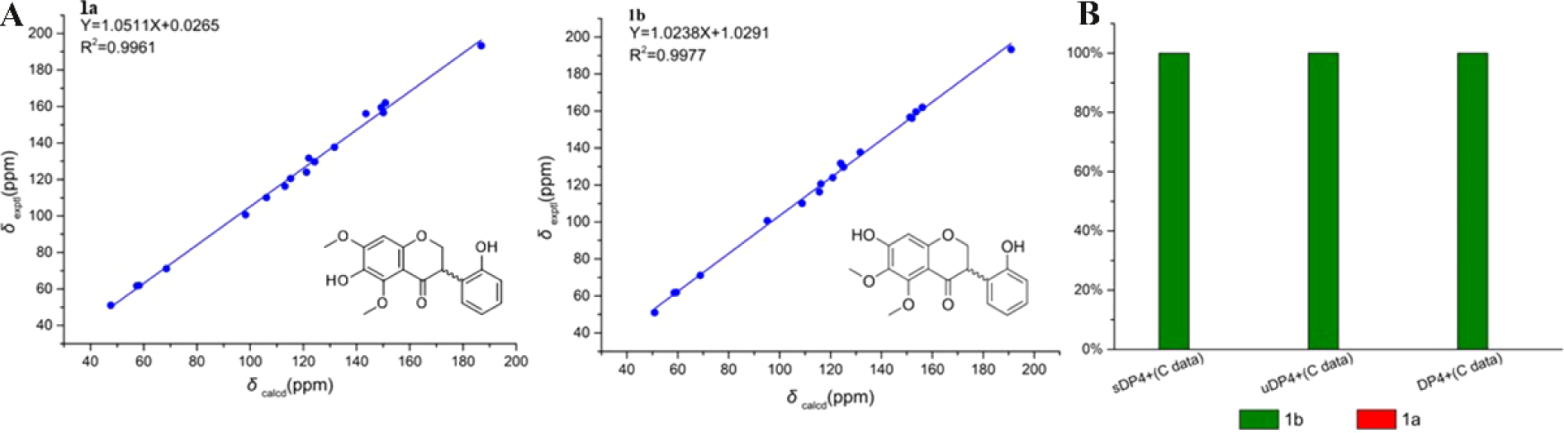
(A) Regression analysis of experimental versus calculated ^13^C NMR chemical shifts of suaeglaucin C (**1a**) and **1**/**2** (**1b**); (B) DP4+ probability for **1a**/**1b**.

**Figure 5:**
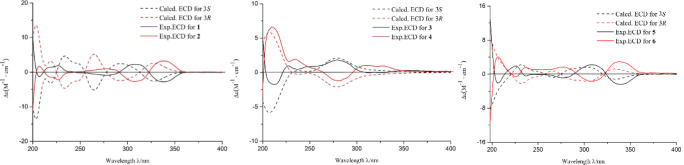
Calculated and experimental ECD spectra of compounds **1**–**6**.

(±)-Agrisquarin B (**3**/**4**) possessed the molecular formula C_17_H_16_O_6_ (*m*/*z* 339.0836 [M + Na]^+^, calcd 339.0839). The ^1^H and ^13^C NMR data (Table 1) of **3**/**4** indicated this compound to be an isoflavanone similar to **1**/**2**, except for the functional groups attached to ring A. The two methoxy groups at δ_H_ 3.80 and 3.91 were linked to C-6 and C-7, respectively, as indicated by HMBC correlations from δ_H_ 3.80 to C-6 (δ_C_ 130.7) and from δ_H_ 3.91 to C-7 (δ_C_ 161.8). The hydroxy group was placed at C-5 by HMBC correlation from 5-OH (δ_H_ 11.59) to C-5 (δ_C_ 155.6). After resolution by a CHIRALCEL IG column (Figure S1), two optically active enantiomers were obtained, and the time-dependent DFT (TDDFT) calculations of their ECD spectra were performed. Details of theoretical ECD calculations are given below (Figure 5). Compared with the experimental curve, the absolute configurations of **3** and **4** were elucidated as 3*S* and 3*R*, respectively. Thus, the structures of **3** and **4** were identified and named (*S*)-agrisquarin B and (*R*)-agrisquarin B, respectively.

The molecular formula of (±)-agrisquarin C (**5**/**6**) was deduced from HRESIMS *m*/*z* 353.0993 ([M + Na]^+^, calcd 353.0996), consistent with C_18_H_18_O_6_Na. The structure of **5**/**6** resembled that of **3**/**4**, as revealed by their closely related ^1^H and ^13^C NMR data. The only difference was that C-5 was substituted by a methoxy group in **5**/**6** instead of a hydroxy group in **3**/**4**, as revealed by an HMBC correlation from δ_H_ 3.90 to δ_C_ 155.1 (C-5). Compound **5**/**6** was also found to be a pair of enantiomers (Figure S1) and was further separated by chiral prep-HPLC. Chemical calculations of their theoretical ECD spectra (Figure 5) established the absolute configurations of **5** and **6** as 3*S* and 3*R*, respectively. Thus, the structures of **5** and **6** were identified and named (*S*)-agrisquarin C and (*R*)-agrisquarin C, respectively.

Agrisquarin D (**7**) has a molecular formula of C_17_H_12_O_6_ based on HRESI data with *m*/*z* ion of 311.0562 for [M – H]^−^ (calcd. 311.0561). The MS data, in combination with 1D and 2D NMR spectra (Table 2), suggested a C_16_ coumaronochromone skeleton having a 5,6,7-trisubstituted A-ring and a 2′-oxygenated B-ring. Further spectroscopic analysis indicated a structure similar to that of cristatone I,^21^ differing in the presence of an additional methoxy group (δ_H_ 3.54; δ_C_ 59.0) in **7**. Based on the HMBC spectrum shown in Figure 3, the methoxy group was attached to C-7 (δ_C_ 162.8). Eventually, the structure of **7** was illustrated as depicted in Figure 1.

Compound **8** was isolated as yellow oil. The HREIMS showed an [M + Na]^+^ ion at *m*/*z* 335.0524 (calcd. 335.0526), corresponding to the molecular formula C_17_H_12_O_6_. The ^1^H NMR spectrum of **8** showed diortho-substituted aromatic protons at δ_H_ 7.71 (1H, dd, *J* = 7.2, 1.2 Hz, H-3′), δ_H_ 7.41 (1H, td, *J* = 7.2, 1.4 Hz, H-4’), δ_H_ 7.44 (1H, td, *J* = 7.2, 1.2 Hz, H-5′), and δ_H_ 8.00 (1H, dd, *J* = 7.2, 1.4 Hz, H-6’), a penta-substituted benzene aromatic proton at δ_H_ 6.94 (1H, s, H-8), and two methoxy signals at δ_H_ 3.79 and 3.85 (each 3H, s, 5-OMe, 6-OMe). The ^13^C NMR spectrum showed a total of 17 carbon signals, including two methoxy signals, five methine, and ten quaternary carbon signals due to a coumaronochromone skeleton. The above NMR features were very similar to those of suaeglaucin A,^22^ with the obvious distinction arising from the absence of a methoxy signal attached to C-8 in **8**, which was replaced by an aromatic proton at δ = 6.94 (1H, s, H-8). This was confirmed by the HMBC correlations from H-8 (6.94, 1H, s) to C-6 (140.0), C-7 (151.0), C-9 (155.7), and C-10 (110.9). Therefore, compound **8** was identified as shown in Figure 1 and named agrisquarin E.

Compound **9** was isolated as a yellow oil. It has the same molecular formula as **8**, which was established from the quasi-molecular ion peak C_17_H_12_O_6_ at *m*/*z* 335.0524 (calcd 335.0526) [M + Na]^+^ in the HRESIMS. The ^1^H NMR spectrum revealed signals for six aromatic protons in the benzofuran ring (δ_H_ 8.09, 1H, s, H-2), A-ring (δ_H_ 6.29, 1H, s, H-8), and B-ring (δ_H_ 7.54, 1H, dd, *J* = 7.3, 1.4 Hz, H-3′; 7.36, 1H, td, *J* = 7.3, 1.4 Hz, H-4’; 7.34, 1H, td, *J* = 7.3, 1.4 Hz, H-5′; and 7.89, 1H, dd, *J* = 7.3, 1.4 Hz, H-6’), a methylenedioxy group at δ_H_ 5.96 (2H, s), and a methoxy group at δ_H_ 3.67 (3H, s, 5-OMe). The ^13^C NMR spectrum (Table 2) exhibited 17 carbon signals, consisting of a methoxyl (δ_C_ 59.9), a methylene (δ_C_ 101.4), six methines (δ_C_ 93.3, 111.6, 121.8, 124.0, 125.1, and 150.4), and nine quaternary carbons (δ_C_ 108.7, 123.6, 125.1, 129.6, 142.2, 154.7, 154.9, 160.4, and 190.5), suggesting that **9** was an aroylbenzofuran formed via a C-ring cleavage from a coumaronochromone skeleton. A meticulous analysis of the NMR data indicated that **9** was related to **8** (Figure 1), characterized by the conversion of the A-ring substituents at C-6 and C-7 into a methylenedioxy group. This was further confirmed by the HMBC cross-peaks from H-2 (δ_H_ 8.09) to C-3 (δ_C_ 123.6)/C-4 (δ_C_ 190.5)/C-1’ (δ_C_ 125.1)/C-2’ (δ_C_ 154.9), H-6’ (δ_H_ 6.29) to C-3 (δ_C_ 123.6), H-5′ (δ_H_ 7.34) to C-1’ (δ_C_ 125.1)/C-3′ (δ_C_ 111.6), 9-OH (δ_H_ 11.62) to C-10 (δ_C_ 108.7)/C-9 (δ_C_ 160.4)/C-8 (δ_C_ 93.3), 5-OMe (δ_H_ 3.67) to C-5 (δ_C_ 142.4), and OCH_2_O (δ_H_ 5.96) to C-7 (δ_C_ 154.7)/C-6 (δ_C_ 129.6) (Figure 2). From the above evidence, the structure of **9** was established and named agrisquarin F.

Compound **10** was obtained as a colorless oil, and the molecular formula was determined as C_17_H_12_O_7_ based on the HRESIMS ion at *m*/*z* 351.0473 [M + Na]^+^ (calcd for 351.0475). The ^1^H NMR spectrum revealed signals for an aromatic proton at δ_H_ 6.29 (1H, s, H-8), a methylenedioxy group [δ_H_ 5.91 and 5.93 (2H, each s, H_2_-1’’)], and a methoxy group [δ_H_ 4.00 (3H, s, 5-OMe)] in ring A, a hydroxy group [δ_H_ 4.63 (1H, s, 3-OH)] in ring C, and four aromatic protons [δ_H_ 6.90 (1H, d, *J* = 8.1 Hz, H-3′), 7.24 (1H, t, *J* = 8.0 Hz, H-4’), 6.94 (1H, t, *J* = 8.1 Hz, H-5′), and 7.22 (1H, d, *J* = 8.1 Hz, H-6’)] in the 2’-*O*-substituted ring B. The ^13^C NMR and DEPT spectra (Table 2) exhibited 17 carbon signals, consisting of a methoxyl (δ_C_ 60.3), a methylene (δ_C_ 101.9), six methines (δ_C_ 94.5, 109.3, 110.6, 122.4, 124.1, and 131.2), and nine quaternary carbons (δ_C_, 82.1, 107.4, 126.9, 132.4, 142.3, 155.2, 156.2, 160.4, and 187.9), indicating that **10** was a coumaronochromone analog similar to (2*R*,3*S*)-3,7,4’-trihydroxy-5-methoxycoumaronochromone.^23^ Comparison of the NMR data between them revealed the absence of two hydroxy groups at C-8 and C-4’, and an additional methylenedioxy (δ_H_ 5.91 and δ_C_ 101.9) observed in **10**. The methylenedioxy was positioned at C-6/C-7 by HMBC correlations from OCH_2_O (δ_H_ 5.91) to C-7 (δ_C_ 155.2) and C-6 (δ_C_ 132.4). The ROESY correlation between H-2 (δ_H_ 6.28) and 3-OH (δ_H_ 4.63) permitted the two protons to be cofacial. The lack of a cotton effect in the ECD spectrum and a specific rotation approaching zero indicated that compound **10** was racemic. Compound **10** was therefore characterized and given the name agrisquarin G.

Additionally, the structures of the known metabolites identified as irisone A (**11**),^24^ 5-methoxy-6,7-methylenedioxy-2’-hydroxyisoflavone (**12**),^25^ 5,2’-dihydroxy-6,7-dimethoxy isoflavone (**13**),^26^ 2’-hydroxy-5,6,7-trimethoxyisoflavonoid (**14**),^27^ kaempferol (**15**),^28^ quercetin (**16**),^29^ isorhamnetin (**17**),^30^ 2’,5,7-trihydroxy-flavanone (**18**),^31^ quercetin-3-*O*-rutinoside (**19**),^32^ isorhamnetin-3-*O*-rutinoside (**20**),^33^ (3*S*, 5*R*)-dihydroxy-6,7-megastigmadien-9-one (**21**),^34^ grasshopper ketone (**22**),^35^ dehydrovomifoliol (**23**),^36^ *N*-*trans*-feruloylmethoxytyramine (**24**),^37^ *N*-*trans*-cinnamoyltyramine (**25**),^38^ ceplignan (**26**),^39^ avellanedae A (**27**),^40^ monogynol A (**28**),^41^ 16-hydroxyolean-12-ene-3,11-dione (**29**),^42^ hennadiol (**30**),^43^ salicylic acid (**31**),^44^ *p*-hydroxybenzoic acid (**32**),^45^ 3,4-dihydroxybenzoic acid (**33**),^46^ vanillic acid (**34**),^47^ scopoletin (**35**),^48^ scoparone (**36**),^49^ 1*H*-indole-3-carboxaldehyde (**37**),^50^ indole-3-carboxylic acid (**38**),^51^ ferulic acid (**39**),^52^ orychophragine A (**40**),^53^ trigonelline (**41**),^54^ uridine (**42**),^55^ uracil (**43**),^56^ (6*R*, 9*S*)-roseoside (**44**),^57^ 5,7-dihydroxy chromone (**45**),^58^ and cinnamic acid (**46**)^59^ were determined by comparing their NMR data to those previously reported in literature.

### 3.2 Anti-Inflammatory Activity and SAR

All of the new and selected known compounds were evaluated for their inhibitory effects on NO production in LPS-induced RAW264.7 cells by the Griess reaction. L-NMMA (52.01 ± 1.96%) was selected as the positive control. Results showed that compounds **1**–**10**, **21**–**26**, **40**, **41**, **44**, and **45** displayed inhibitory effects on NO production of RAW 264.7 cells, with inhibition rates ranging from 1.46 ± 0.30% to 88.58 ± 1.06% (Figure 6 and Table 3). Among them, compounds **5** and **21** exhibited inhibitory effects (Table 4) comparable to or stronger than the positive control drug, with IC_50_ values of 40.52 ± 1.06 μM and 19.27 ± 0.24 μM, respectively (L-NMMA, IC_50_ = 36.83 ± 2.00 μM).

**Figure 6:**
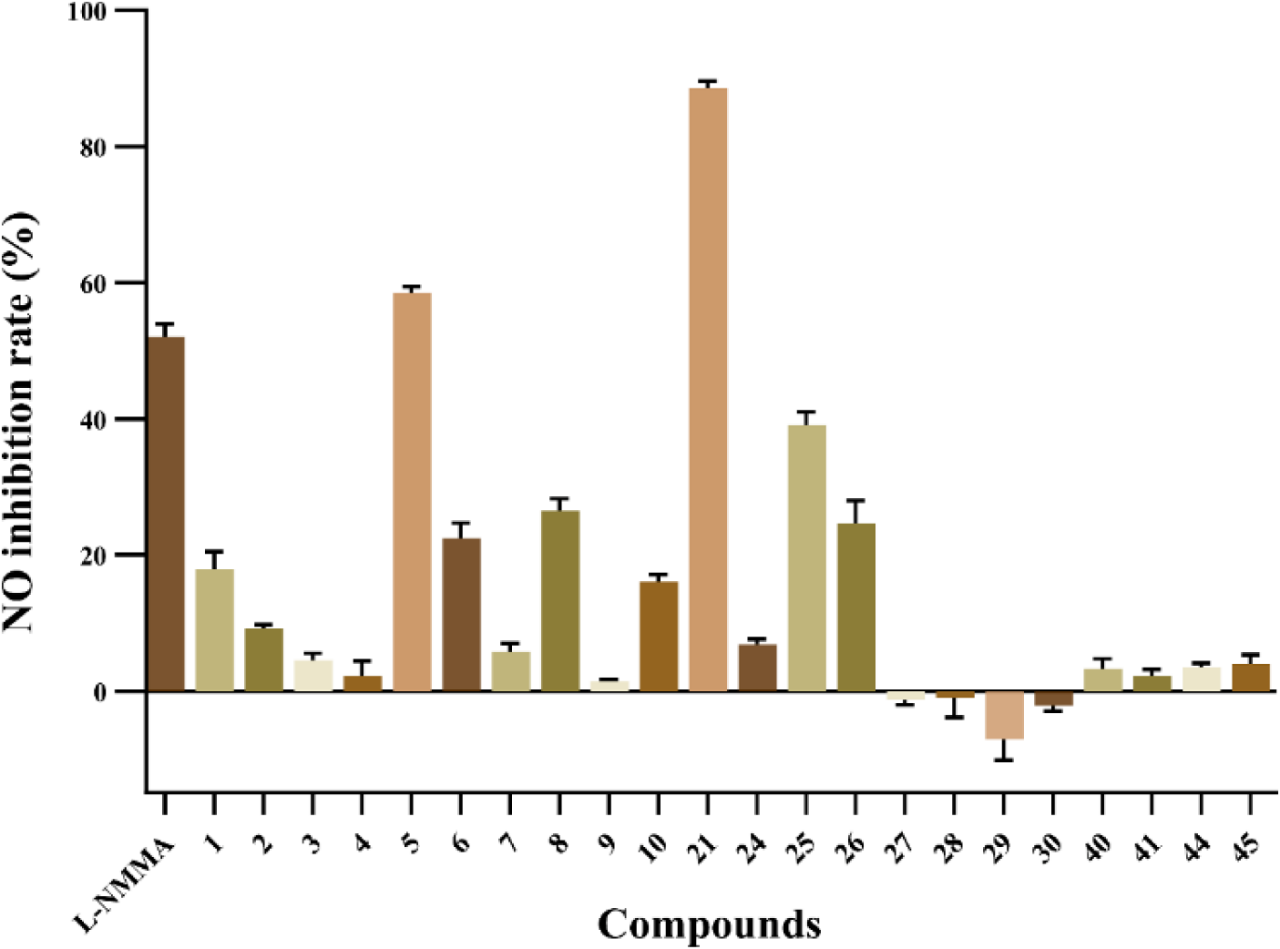
Inhibitory effects of compounds **1**–**10**, **21**–**26**, **40**, **41**, **44**, and **45** at the 50 μΜ level on NO production in LPS-induced RAW 264.7.

**Table 3:** Inhibitory Effects of **1**–**10**, **21**, and **24**–**30** on NO Production in LPS-Induced RAW 264.7

Compd^a^	Inhibition rate (%)	Compd^a^	Inhibition rate (%)
**1** (3*S*)	17.90 ± 2.65	**25**	39.12 ± 1.93
**2** (3*R*)	9.21 ± 0.58	**26**	24.63 ± 3.31
**3** (3*S*)	4.46 ± 1.06	**27**	-1.22 ± 0.80
**4** (3*R*)	2.33 ± 2.09	**28**	-0.95 ± 2.85
**5** (3*S*)	58.58 ± 0.85	**29**	-7.12 ± 3.06
**6** (3*R*)	22.40 ± 2.34	**30**	-2.10 ± 0.87
**7**	5.77 ± 1.24	**40**	3.24 ± 1.54
**8**	26.51 ± 1.80^c^	**41**	2.32 ± 0.82
**9**	1.46 ± 0.30	**44**	3.46 ± 0.63
**10**	16.10 ± 1.04	**45**	3.96 ± 1.43
**21**	88.58 ± 1.06	L-NMMA^b^	52.01 ± 1.96
**24**	6.82 ± 0.86		

a Concentration at 50 μM.

b Positive control substance in RAW 264.7.

c Cytotoxic effect was observed.

**Table 4:** Inhibitory Effects of the Isolates on LPS-Activated NO Production in RAW 264.7^a^

Compd	IC_50_ (μM, mean ± SD, *n* = 3)
**5**	40.52 ± 1.06
**21**	19.27 ± 0.24
L-NMMA^b^	36.83 ± 2.00

a Only compounds with observable inhibitory effects (IC_50_ < 50 μM) are listed.

b Positive control substance in RAW 264.7.

As shown in Table 4, the inhibitory rate (%) for (*S*)-agrisquarins A (**1**), B (**3**), and C (**5**) (17.90 ± 2.65, 4.46 ± 1.06, and 58.58 ± 0.85) was approximately 2–3 times stronger than that of their C-3 enantiomers (*R*)-agrisquarins A (**2**), B (**4**), and C (**6**) (9.21 ± 0.58, 2.33 ± 2.09, and 22.40 ± 2.34), respectively. Additionally, among (*S*)-agrisquarins A (**1**), B (**3**), and C (**5**), compound **5**, with methoxy groups at C-5, C-6, and C-7, exhibited the highest NO inhibitory rate (58.58 ± 0.85%); compound **1** (methoxy groups at C-5 and C-6) showed a moderate inhibitory rate (17.90 ± 2.65%); and compound **3** (methoxy groups at C-6 and C-7) exhibited the lowest activity (4.46 ± 1.06%). A similar structure–activity relationship (SAR) was also observed for (*R*)-agrisquarins A (**2**), B (**4**), and C (**6**), with compound **6** showing about 3 and 10 times higher levels than compounds **2** and **4**, respectively. These results allowed us to speculate on the SAR of these dihydroisoflavones: the stereochemistry at C-3 and the position of methoxy substitution are closely related to their anti-inflammatory activity.

## 4 Conclusions

In conclusion, previous studies provided evidence supporting the benefits of A. squarrosum as a nutritional food or a functional feed source enhancing the ruminant’s immune response and anti-inflammatory capacity. However, the phytochemicals from this plant remain unclear. In this study, ten undescribed isoflavanone derivatives (**1**–**10**), together with 36 known analogs (**11**–**46**) were isolated from A. squarrosum. All compounds were isolated from this plant for the first time, except **11**, **19**, and **20**. The absolute configurations of **1**–**6** were determined by spectroscopic analyses and DFT calculations of their ECD spectra. The structure of suaeglaucin C, which has multiple substituents in ring A and cannot be elucidated by 2D-NMR, was revised facilely by a 1D-NMR-based method and was further confirmed by subsequent quantum chemical calculations.

Despite the phytochemicals from the seeds of A. squarrosum showed almost no inhibitory effects on NO production of RAW 264.7 cells, the results of chemicals derived from the aerial parts of the plant were promising, which supported the claimed benefits of A. squarrosum as a dietary supplement for ruminants in terms of enhancing anti-inflammatory capacity. Notably, compounds **5** and **21** exhibited anti-inflammatory effects comparable to or stronger than those of the positive control drug. Interestingly , the structure–activity relationship of dihydroisoflavones indicated that the stereochemistry at C-3 might play a key role for their anti-inflammatory activity. These findings establish a molecular and bioactivity foundation for A. squarrosum’s potential, especially in the arid or alpine regions, as a functional feed source aimed at inflammation prevention.

## Supplementary Material



## Data Availability

Data are contained within the article or Supporting Information.

## Notes

Computational details for compounds **1**, **3**, **5**, and suaeglaucin C; Table S1: energy analysis for conformers of **1**, suaeglaucin C, **3**, and **5**; Table S2: DP4+ probabilities of **1** and suaeglaucin C; Figure S1: the chiral HPLC separation chromatograms of compounds **1**/**2**, **3**/**4**, and **5**/**6**.; Figures S2–S36: NMR and HRESIMS spectra of **1**–**10** (PDF)
