# Supplementary material for: Phytochemicals from a Desert Crop, Sand Rice (Agriophyllum squarrosum), and Their Inflammatory Activity
Source: ACS Omega. 2025 May 21;10(21):21846–56. doi: 10.1021/acsomega.5c01825 (PMC12138692; doi:10.1021/acsomega.5c01825)
Supplement: Supplementary file 1 [file ao5c01825_si_001.pdf]

Phytochemicals from a Desert Crop, Sand Rice (*Agriophyllum squarrosum*), and Their Inflammatory Activity

Ping Hai, Qiang Li, Hai Yan Jia, Xiao Fei Ma, Yun Qing He, Jin Yang, Xian Yan Li, Zhi Qiang Luo, Mei Ling Yang, Yuan Gao*, Hong Peng Wang*, Jian Yang*

1. The chiral HPLC separation chromatograms of compounds 1/2, 3/4, and 5/6. ..3

2. Computational Details..4

3. NMR and MS spectra of compound 1/2. 6

4. NMR and MS spectra of compound 3/4. 10

5. NMR and MS spectra of compound 5/6. 13

6. NMR and MS spectra of compound 7. 16

7. NMR and MS spectra of compound 8. 19

8. NMR and MS spectra of compound 9. 23

9. NMR and MS spectra of compound 10. 26

1. The chiral HPLC separation chromatograms of compounds 1/2, 3/4, and 5/6.

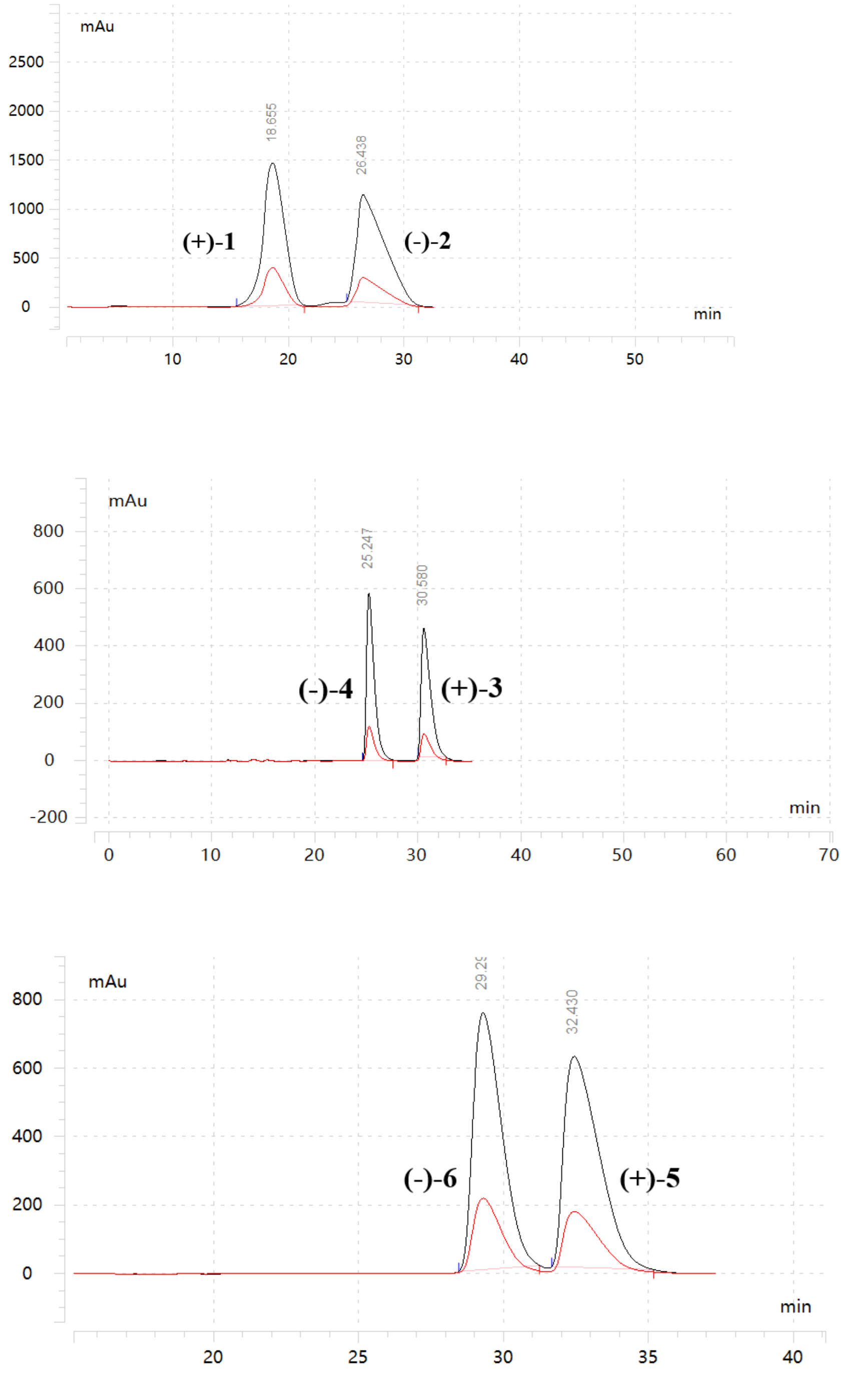

Figure S1 Semipreparative of the mixture of **1**/**2**, **3**/**4**, and **5**/**6** by chiral HPLC (Chiralpak IG, 5 μm, 10 × 250 mm), **1**/**2** [n-hexane/EtOH = 85/15(V/V), 3.0 mL/min]; **3**/**4** [n-hexane/EtOH = 80/20 (V/V), 3.0 mL/min]; **5**/**6** [n-hexane/EtOH = 84/16(V/V), 3.0 mL/min].

2. Computational Details

2.1 Energy analysis for conformers

Conformation searches based on molecular mechanics with MMFF94s force field were performed for **1**, **suaeglaucin C (sua C)**, **3,** and **5** gave 4, 5, 5, and 6 conformers with populations higher than 1%[1], respectively. All these conformers were further optimized by the density functional theory method at the B3LYP/6-31G(d) level in Gaussian 16 program package[2].

Table S1 Energy analysis for conformers of 1, sua C, 3, and 5 at B3LYP/6-31G(d) level in the gas phase.

species	Gibbs free energy	*ΔE* (kcal/mol)	*PE*%
**1f**	-1108.486597	0.000	36.16%
**1g**	-1108.486347	0.157	27.70%
**1a**	-1108.486212	0.242	23.99%
**1b**	-1108.485502	0.687	11.25%
**sua Cb**	-1108.487337	0.000	36.84%
**sua Cd**	-1108.487202	0.084	31.90%
**sua Ce**	-1108.487023	0.197	26.36%
**sua Ch**	-1108.484627	1.700	2.05%
**sua Ci**	-1108.484278	1.919	1.41%
**3g**	-1108.754556	0.000	49.82%
**3c**	-1108.75338	0.737	14.22%
**3b**	-1108.753315	0.778	13.27%
**3d**	-1108.753161	0.875	11.26%
**3h**	-1108.752998	0.977	9.46%
**5i**	-1147.759728	0.000	49.67%
**5c**	-1147.759258	0.294	30.09%
**5d**	-1147.758612	0.700	15.11%
**5g**	-1147.756753	1.866	2.08%
**5a**	-1147.756309	2.145	1.30%
**5h**	-1147.756204	2.211	1.16%

2.2 NMR calculation details for the compound 1 and Sua C

These optimized conformers were subjected to NMR calculation. Gauge-Independent Atomic Orbital (GIAO) calculations of ^{13}C NMR of the conformers were accomplished by density functional theory (DFT) at mPW1PW91/6-311+G(d,p) level in methanol with PCM model[3]. The ^{13}C NMR chemical shift of TMS was calculated in the same level and used as reference. The calculated ^{13}C NMR data of these conformers were averaged according to the Boltzmann distribution theory and their relative Gibbs free energy.

Table S2 The DP4+ probabilities of 1 and sua C

PCM-mPW1PW9/16-311+G(d,p)/ Unsacled Tensors				
**Nuclei**	**sp2?**	**experimental**	**1**	**sua C**
C-4	x	193.3	190.9	186.8
C-9	x	162.0	156.1	150.8
C-7	x	159.5	153.6	149.3
C-5	x	156.1	152.0	143.5
C-2'	x	156.6	151.3	150.1
C-6	x	137.7	131.7	131.7
C-4'	x	129.7	125.1	124.3
C-6'	x	131.7	124.1	122.0
C-1'	x	124.0	120.9	121.2
C-5'	x	120.6	116.2	115.2
C-3'	x	116.3	115.6	113.1
C-10	x	110.1	108.84	106.14
C-8	x	100.6	95.15	98.27
C-2		71.1	68.82	68.51
5-OMe		61.9	59.43	58.22
6-OMe		61.7	58.57	57.34
C-3		51.0	50.85	47.58

2.3 ECD calculation details of compounds 1, 3 and 5

The optimized conformers **1a**, **1b**, **1f**, **1g**, **3b~d**, **3g**, **3h**, **5a**, **5c**, **5d**, **5i**, **5g**, and **5h** were further subjected to theoretical calculation of ECD at B3LYP/6-311+G (2d, p) level in methanol with PCM model by using time-dependent density functional theory (TDDFT). The calculated ECD curves for were generated using SpecDis 1.71[4].

3. NMR and MS spectra of compound 1/2.

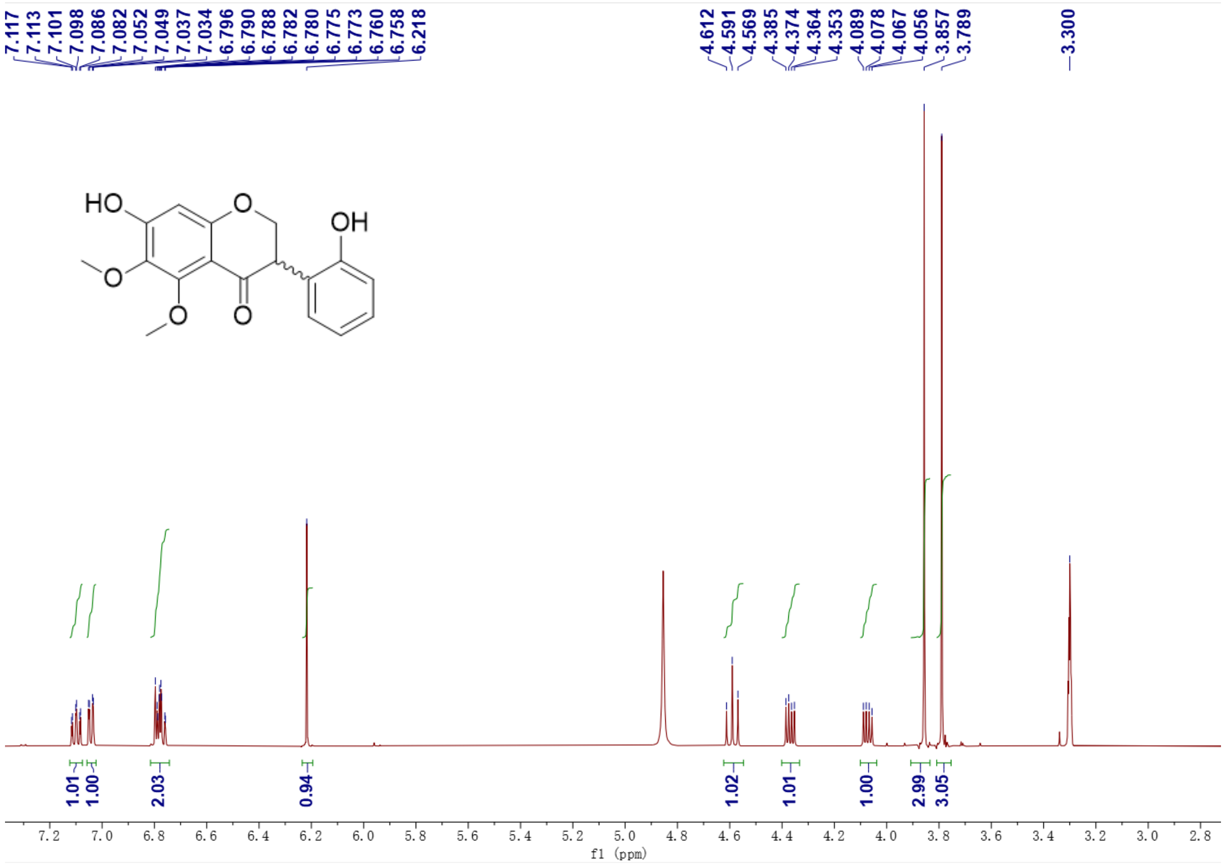

Figure S2 ^{1}H-NMR spectrum (500 MHz) of 1/2 in CD_3OD.

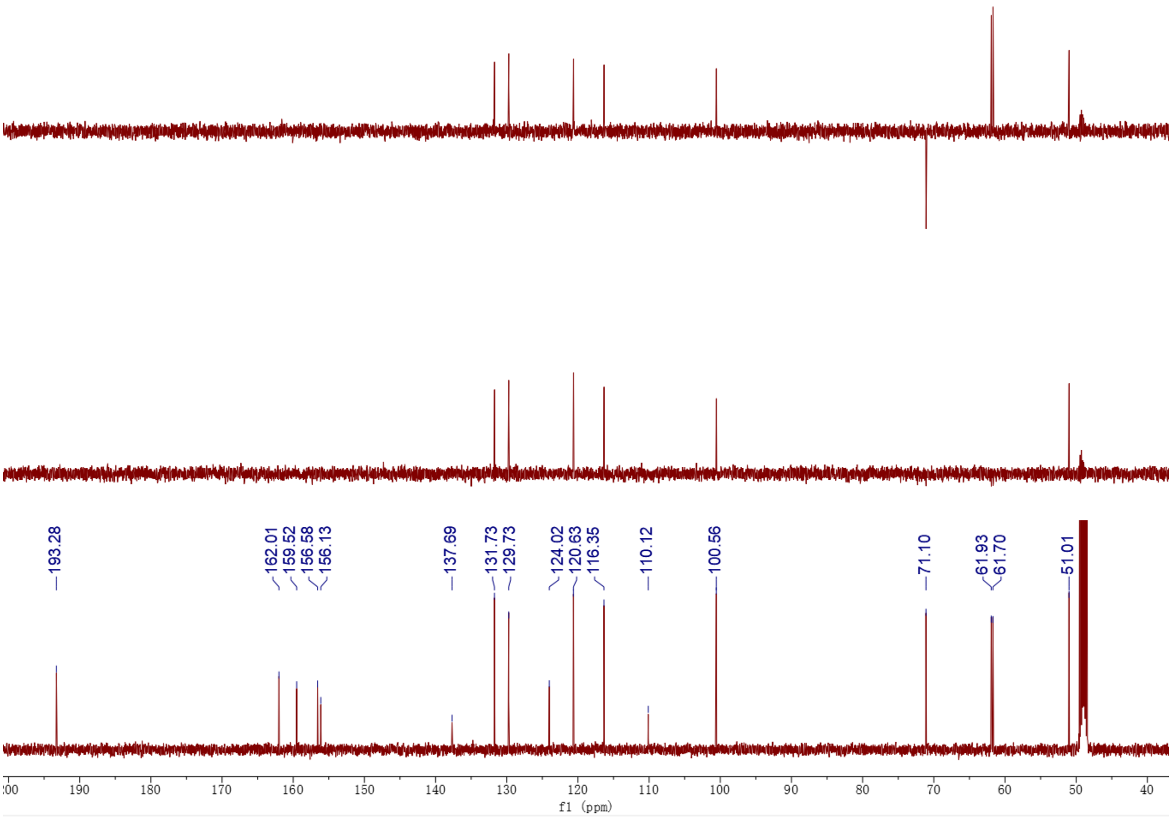

Figure S3 ^{13}C-NMR spectrum (125 MHz) of 1/2 in CD_3OD.

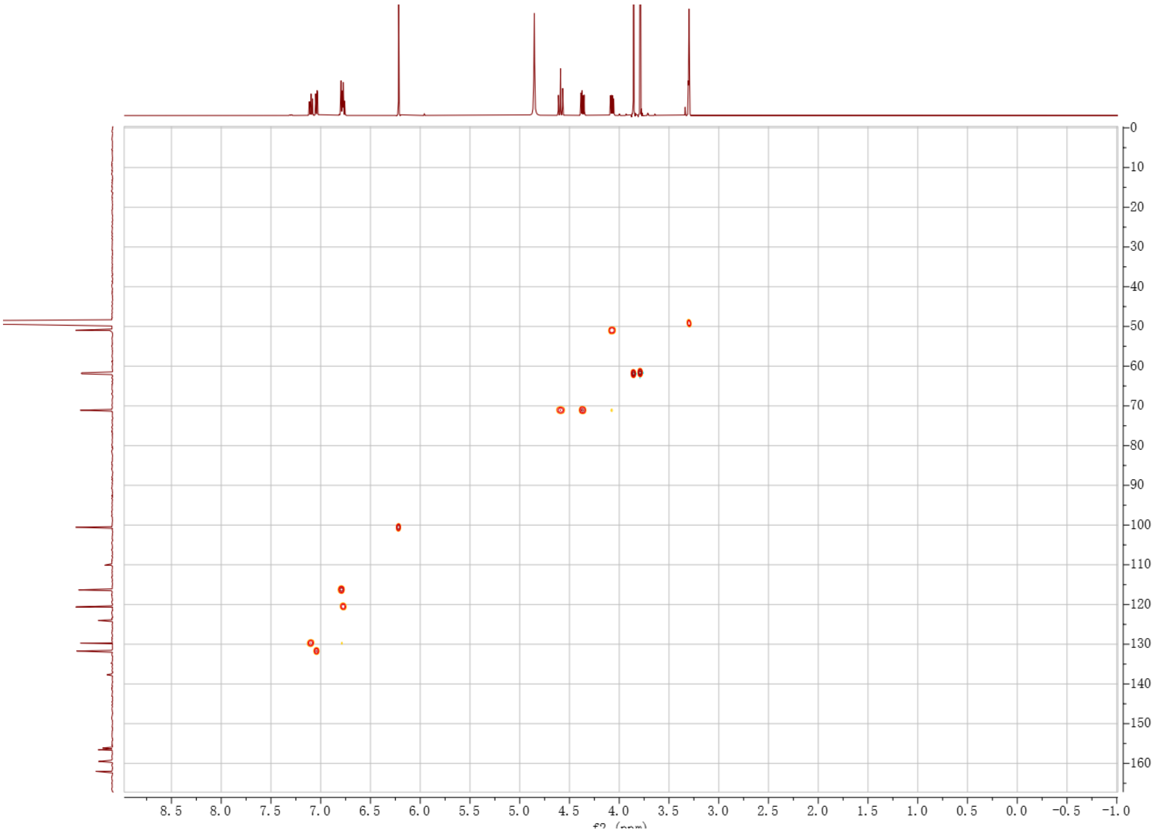

Figure S4 HSQC spectrum (500 MHz) of 1/2 in CD_3OD.

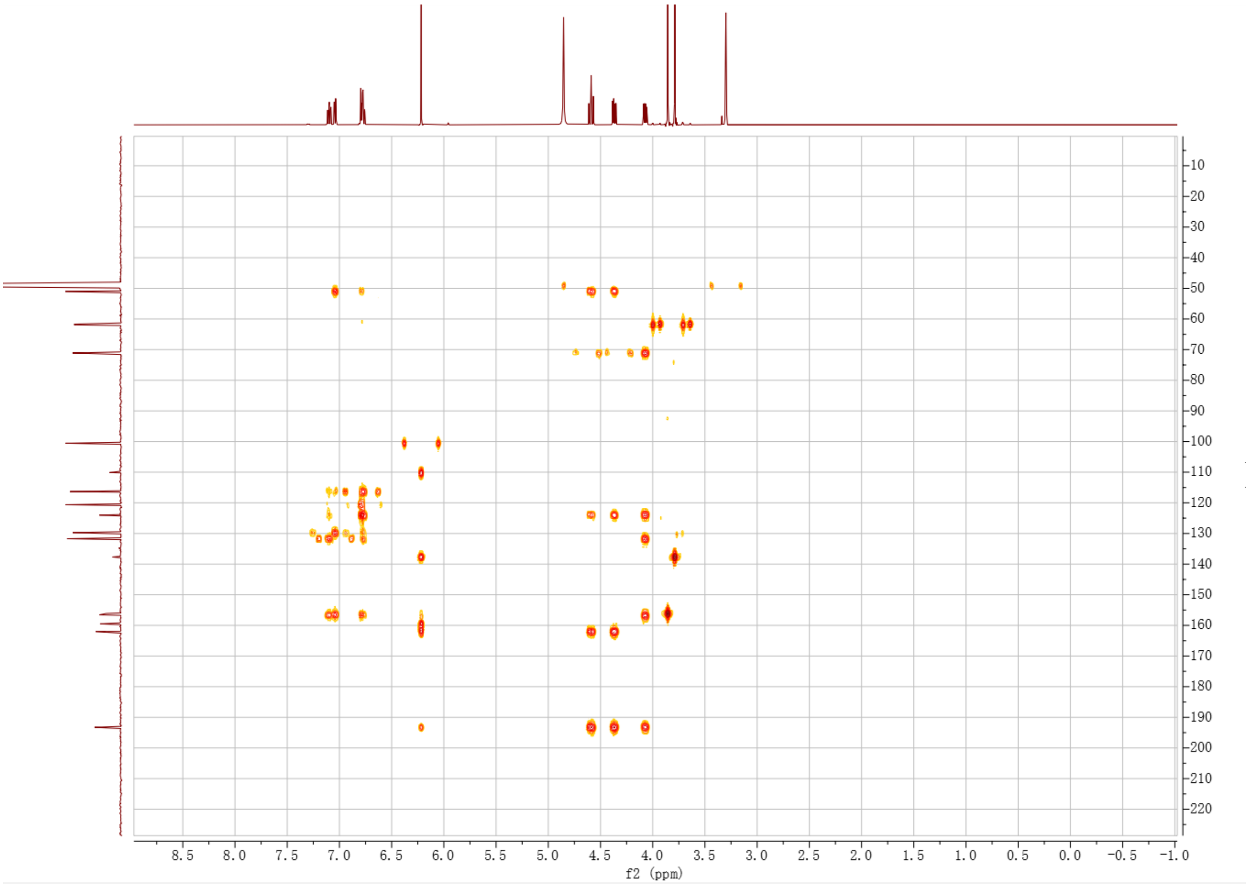

Figure S5 HMBC spectrum (500 MHz) of 1/2 in CD_3OD.

Formula Predictor Report - eas43.lcd

Data File: E:\DATA\2021\0202\eas43.lcd

Elmt	Val.	Min	Max	Elmt	Val.	Min	Max	Elmt	Val.	Min	Max	Elmt	Val.	Min	Max	Use Adduct
H	1	10	100	F	1	0	0	S	2	0	0	Br	1	0	0	H
2H	1	0	0	Na	1	0	0	Cl	1	0	5	Pd	2	0	0	
C	4	5	50	Mg	2	0	0	Co	2	0	0	Ag	1	0	0	
N	3	0	10	Si	4	0	0	Cu	2	0	0	I	3	0	0	
O	2	0	30	P	3	0	0	Se	2	0	0					

Error Margin (ppm): 5
HC Ratio: unlimited
Max Isotopes: all
MSn Iso RI (%): 75.00

DBE Range: -2.0 - 100.0
Apply N Rule: yes
Isotope RI (%): 1.00
MSn Logic Mode: OR

Electron Ions: both
Use MSn Info: yes
Isotope Res: 10000
Max Results: 20

Event#: 2 MS(E-) Ret. Time : 0.240 -> 0.387 Scan# : 38 -> 60

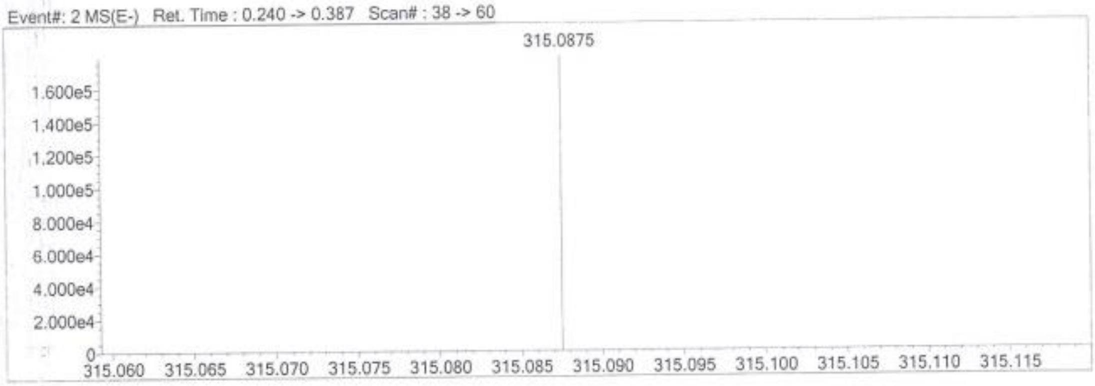

Measured region for 315.0875 m/z

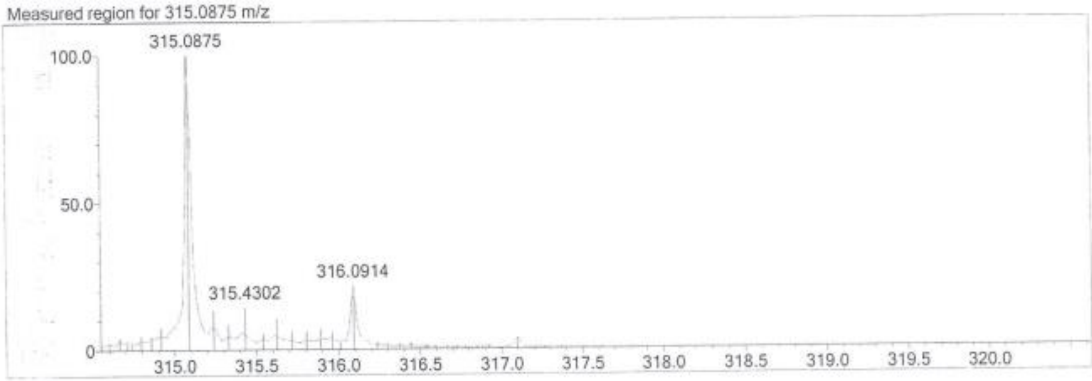

C17 H16 O6 [M-H]- : Predicted region for 315.0874 m/z

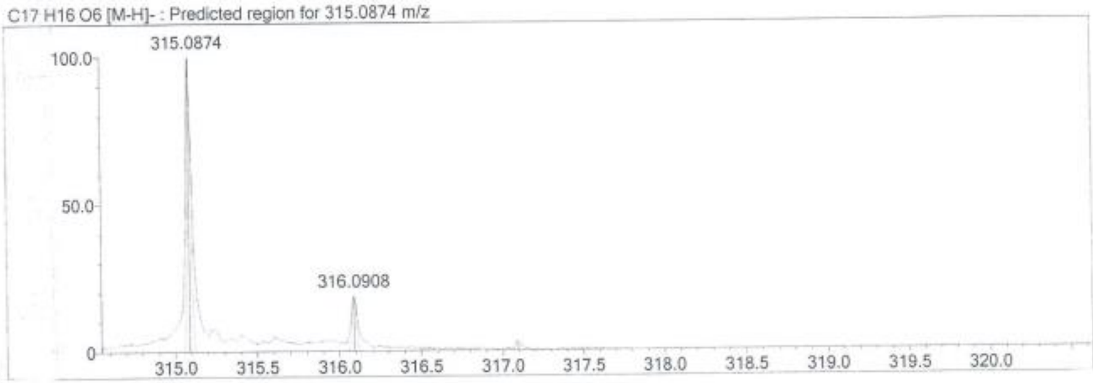

Formula (M)	Ion	Meas. m/z	Pred. m/z	Df. (mDa)	Df. (ppm)	DBE
C17 H16 O6	[M-H]-	315.0875	315.0874	0.1	0.32	10.0

Figure S6 HRESIMS spectrum of 1/2.

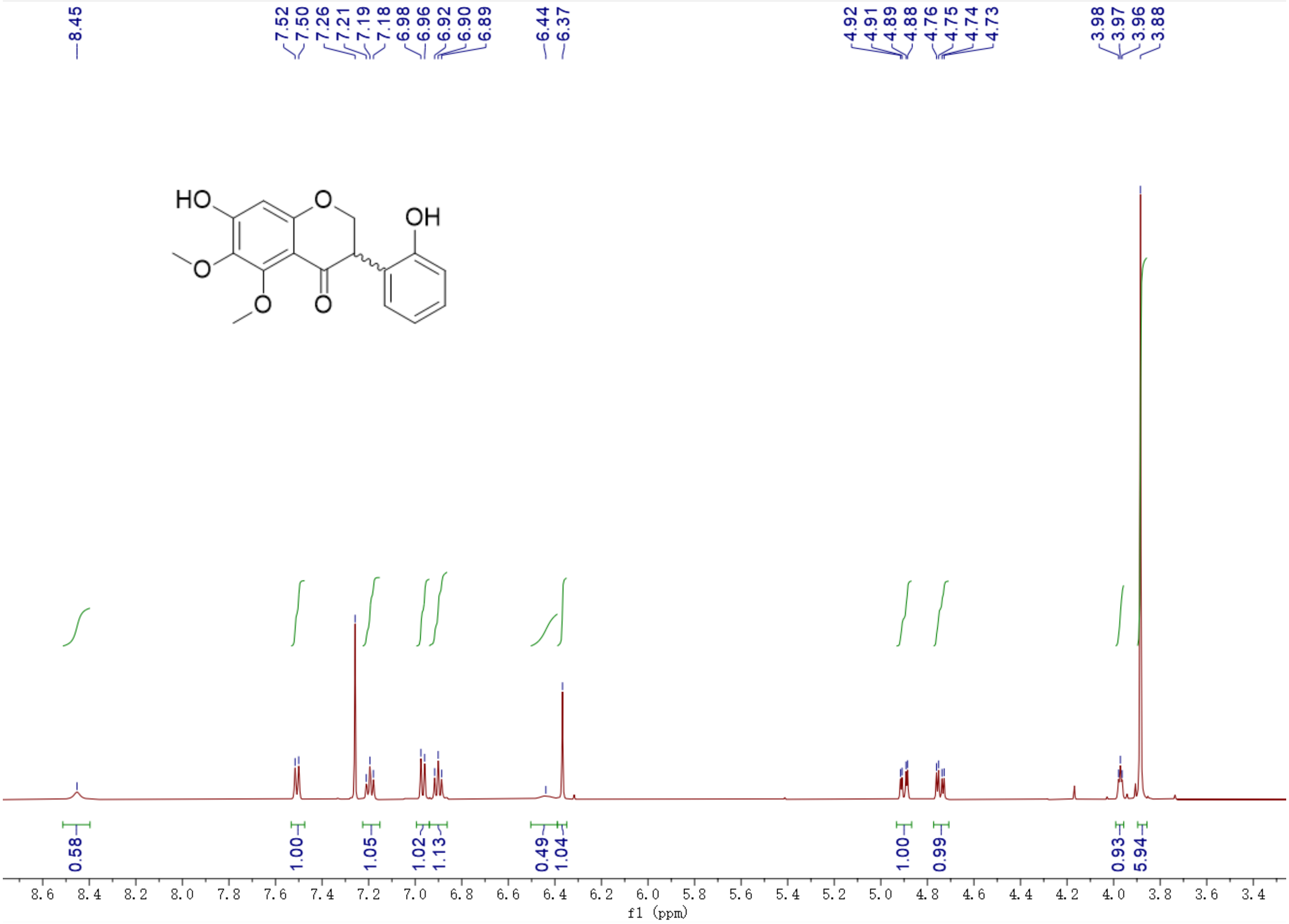

Figure S7 ^{1}H-NMR spectrum (500 MHz) of 1/2 in $CDCl_3$.

4. NMR and MS spectra of compound 3/4.

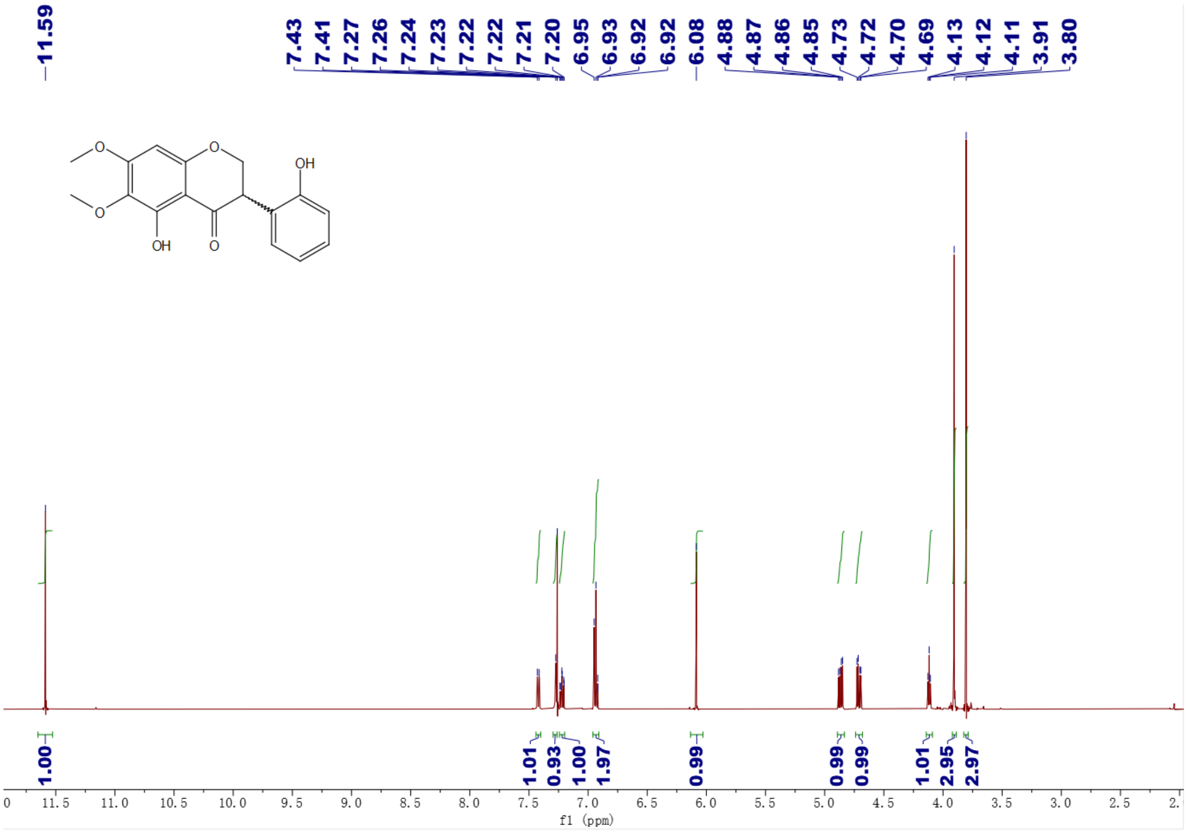

Figure S8 ^{1}H-NMR spectrum (500 MHz) of 3/4 in $CDCl_3$.

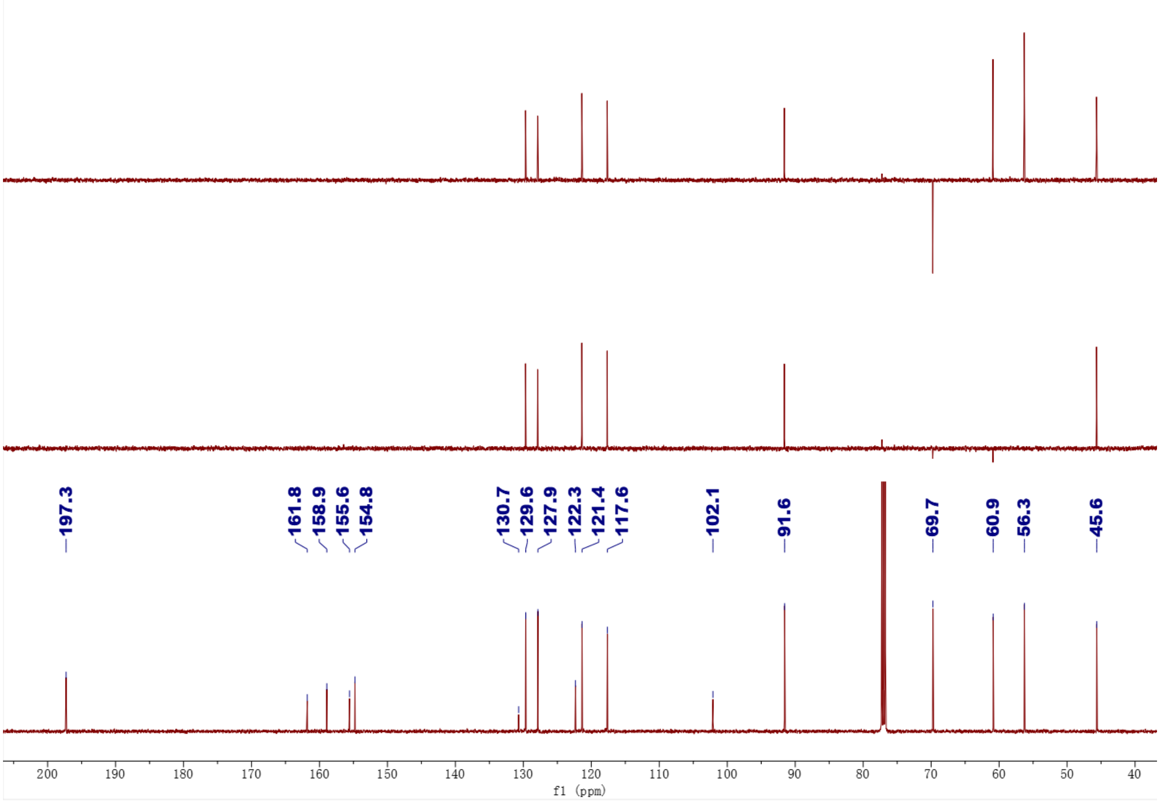

Figure S9 ^{13}C-NMR spectrum (125 MHz) of 3/4 in $CDCl_3$.

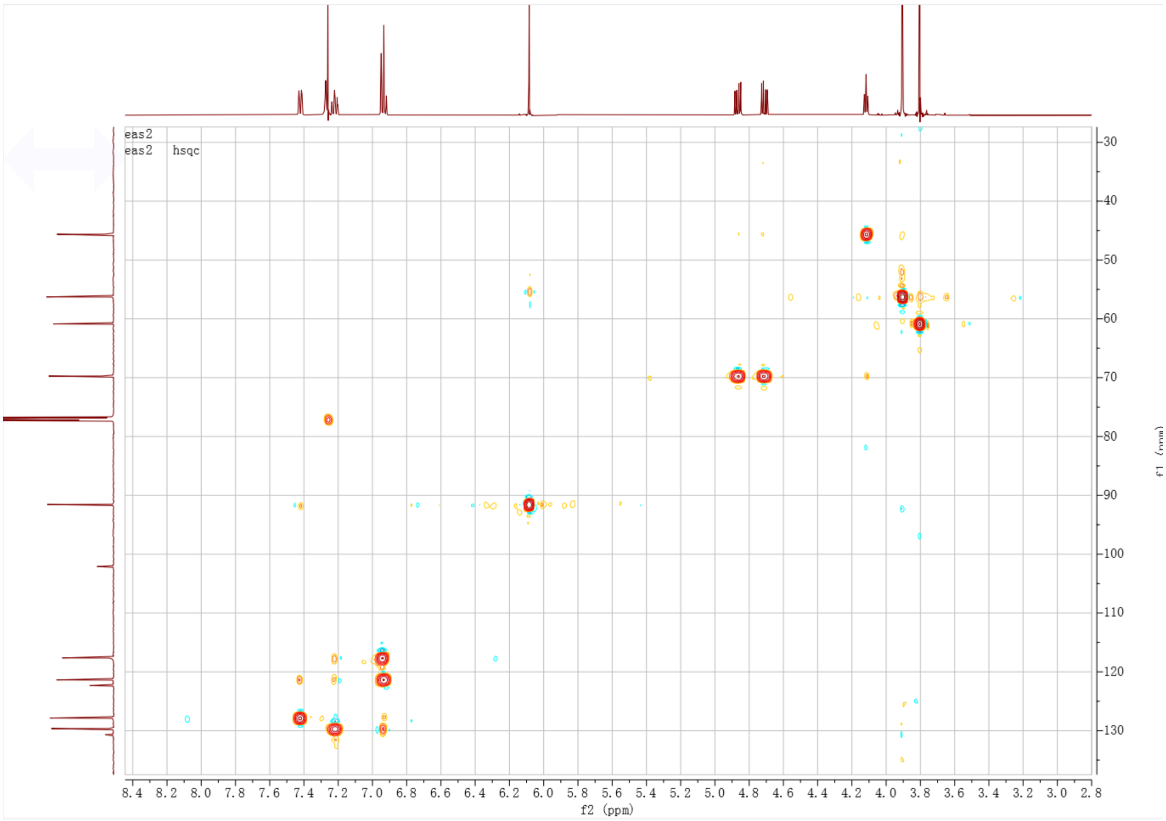

Figure S10 HSQC spectrum (500 MHz) of 3/4 in $CDCl_3$.

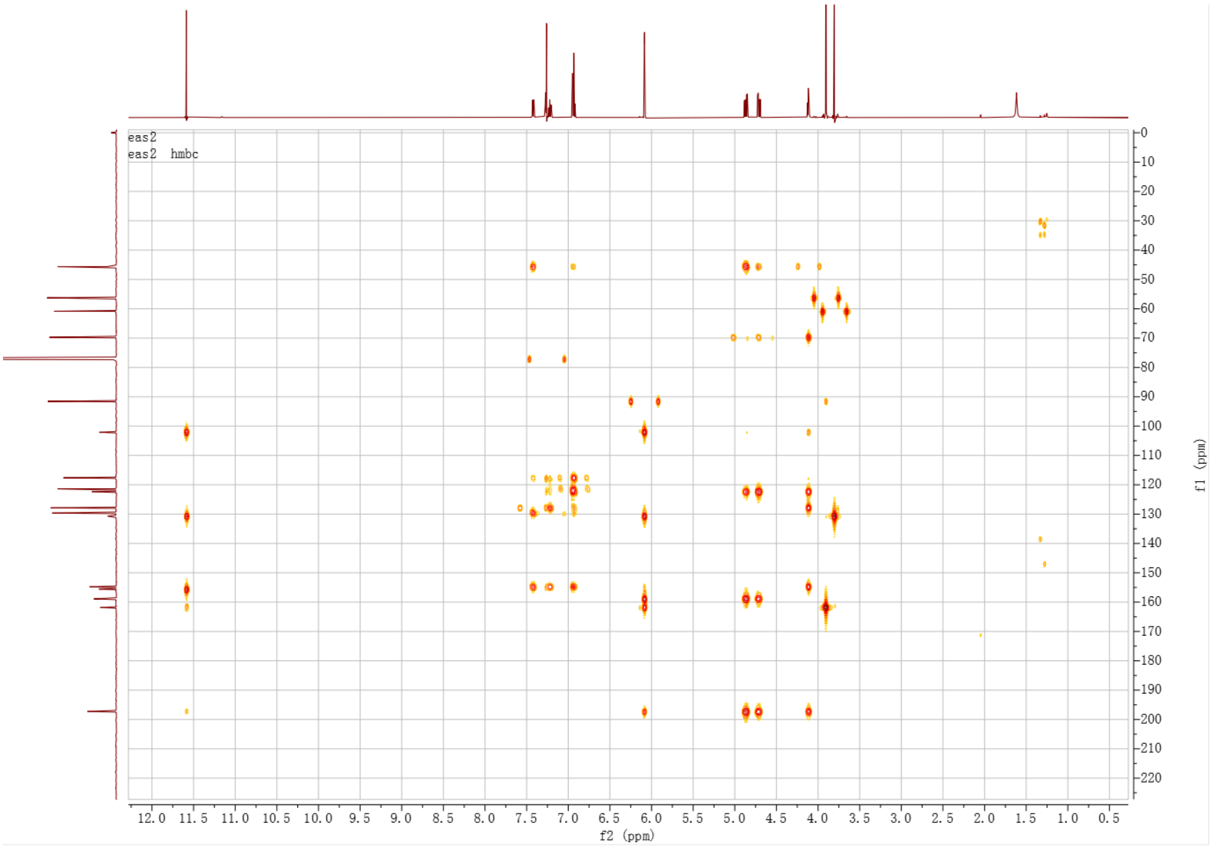

Figure S11 HMBC spectrum (500 MHz) of 3/4 in $CDCl_3$.

Formula Predictor Report - eas2.lcd

Data File: E:\DATA\2020\1021\eas2.lcd

Elmt	Val.	Min	Max	Elmt	Val.	Min	Max	Elmt	Val.	Min	Max	Elmt	Val.	Min	Max	Use Adduct
H	1	10	110	F	1	0	0	S	2	0	0	Br	1	0	0	Na
2H	1	0	0	Na	1	0	0	Cl	1	0	0	Pd	2	0	0	
C	4	5	50	Mg	2	0	0	Co	2	0	0	Ag	1	0	0	
N	3	0	10	Si	4	0	0	Cu	2	0	0	I	3	0	0	
O	2	0	40	P	3	0	0	Se	2	0	0					

Error Margin (ppm):	5	DBE Range:	-2.0 - 100.0	Electron Ions:	both
HC Ratio:	unlimited	Apply N Rule:	yes	Use MSn Info:	yes
Max Isotopes:	all	Isotope RI (%):	1.00	Isotope Res:	10000
MSn Iso RI (%):	75.00	MSn Logic Mode:	OR	Max Results:	10

Event#: 1 MS(E+) Ret. Time : 0.440 -> 0.613 Scan# : 67 -> 93

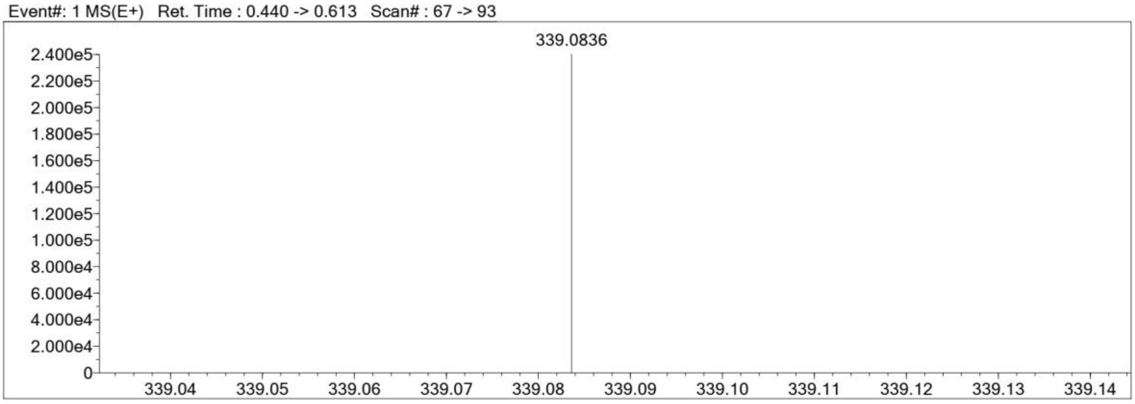

Measured region for 339.0836 m/z

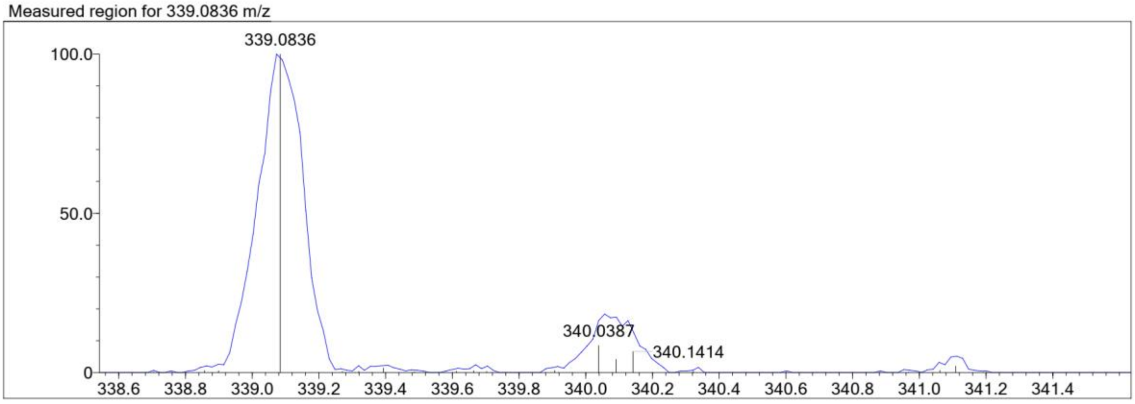

C17 H16 O6 [M+Na]+ : Predicted region for 339.0839 m/z

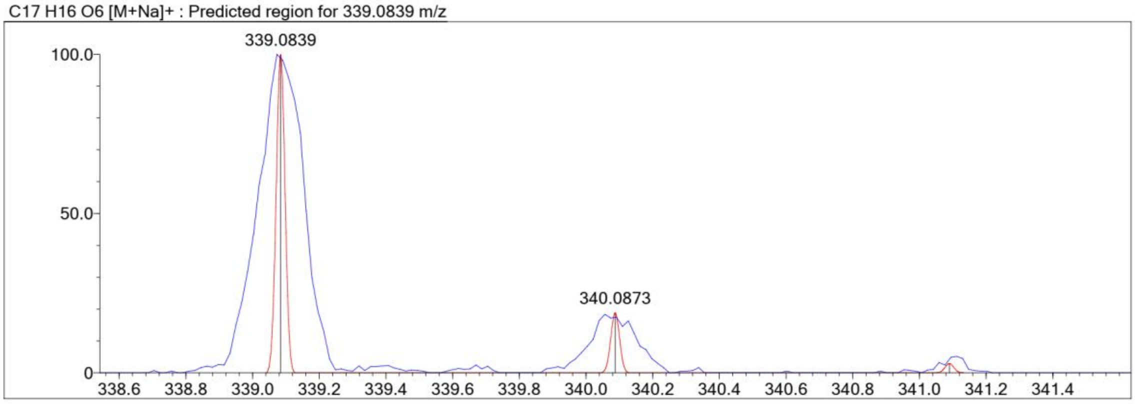

Formula (M)	Ion	Meas. m/z	Pred. m/z	Df. (mDa)	Df. (ppm)	DBE
C17 H16 O6	[M+Na]+	339.0836	339.0839	-0.3	-0.88	10.0

Figure S12 HRESIMS spectrum of 3/4.

5. NMR and MS spectra of compound 5/6.

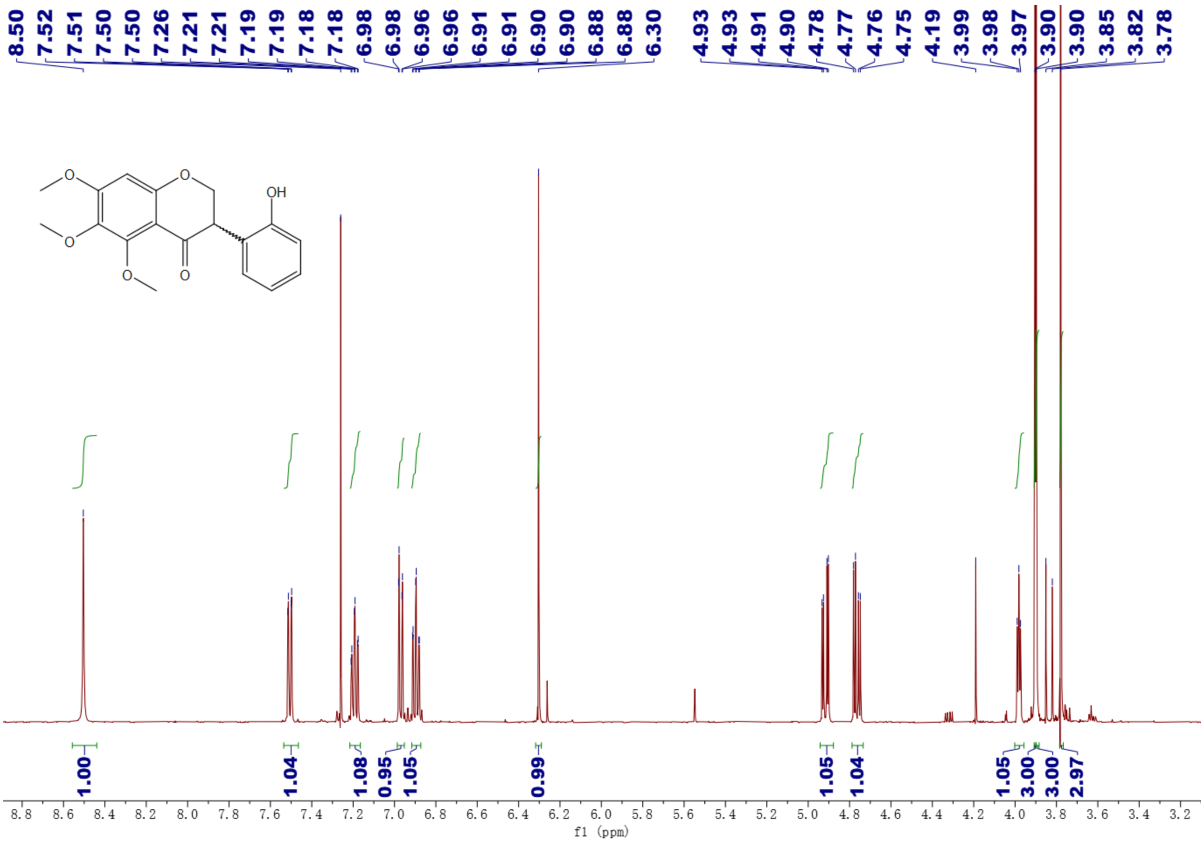

Figure S13 ^{1}H-NMR spectrum (500 MHz) of 5/6 in $CDCl_3$.

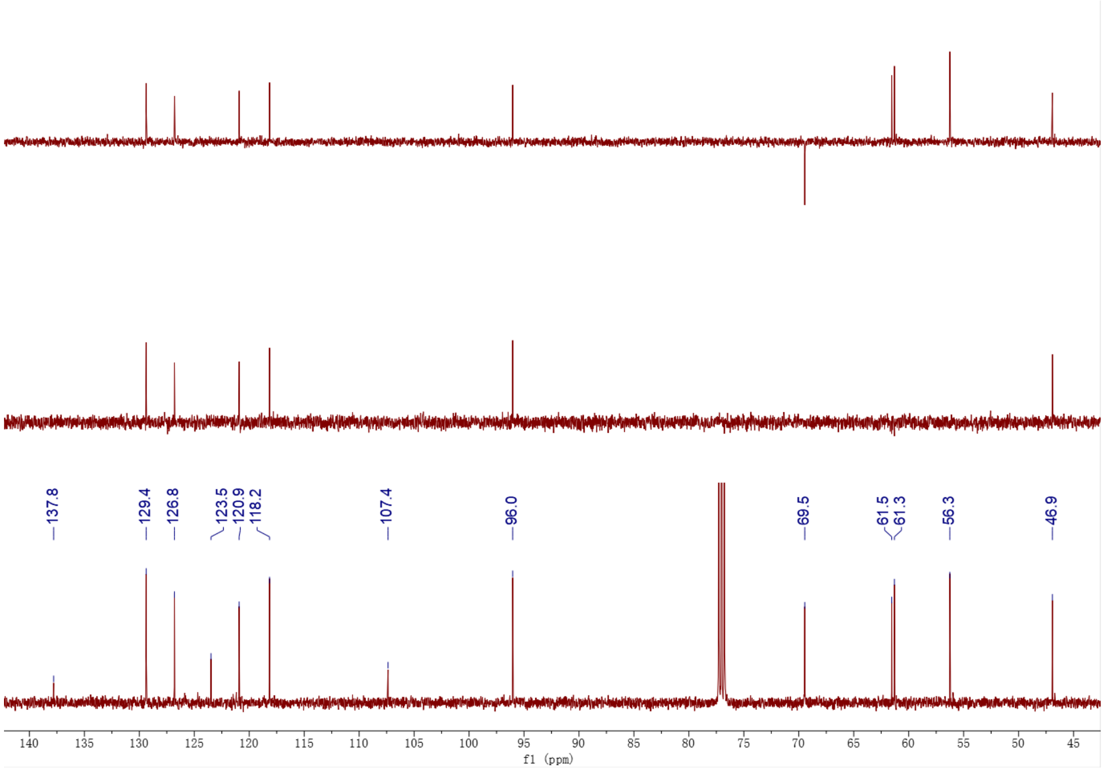

Figure S14 ^{13}C-NMR spectrum (125 MHz) of 5/6 in $CDCl_3$.

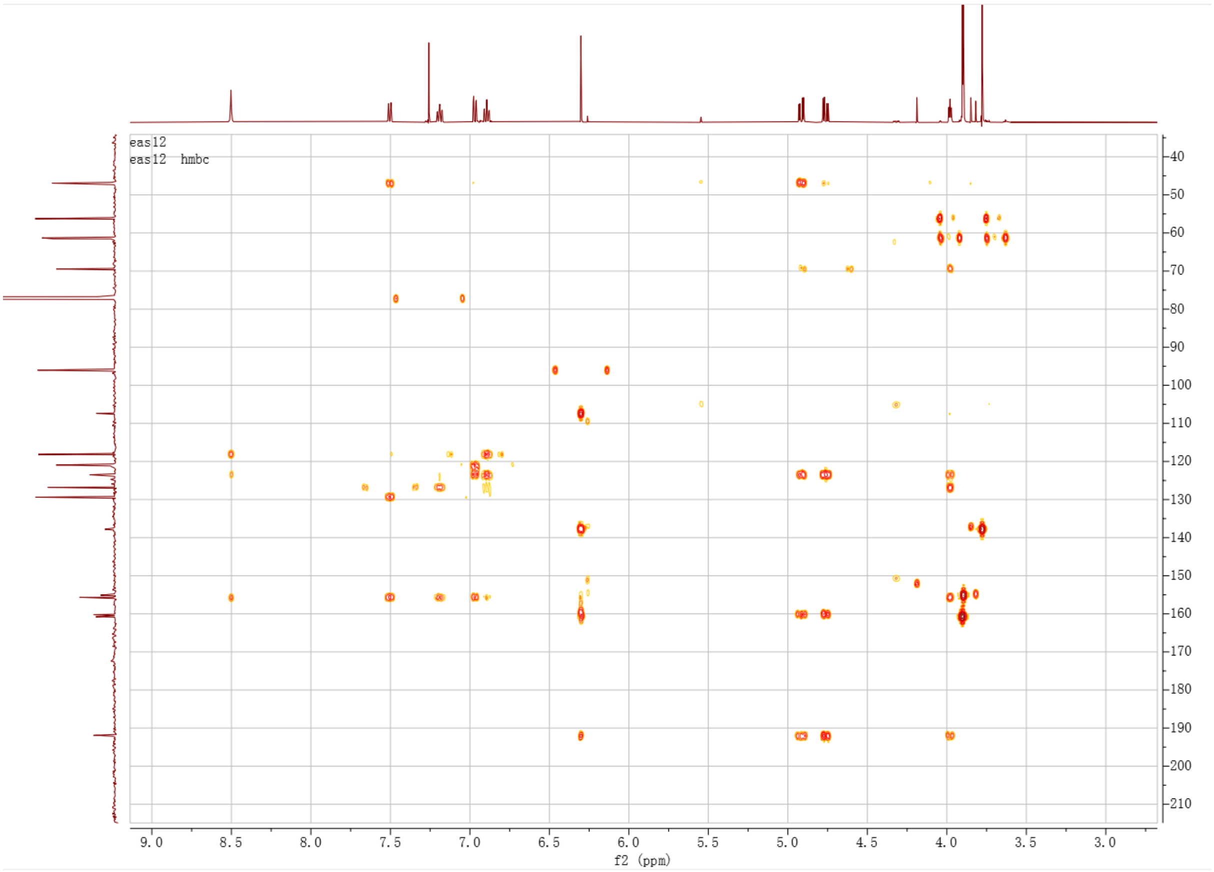

Figure S15 HMBC spectrum (500 MHz) of 5/6 in $CDCl_3$.

Data File: E:\DATA\2020\1124\eas12.lcd

Elmt	Val.	Min	Max	Elmt	Val.	Min	Max	Elmt	Val.	Min	Max	Elmt	Val.	Min	Max	Use Adduct
H	1	5	100	F	1	0	0	S	2	0	0	Br	1	0	0	Na
2H	1	0	0	Na	1	0	0	Cl	1	0	5	Pd	2	0	0	
C	4	5	50	Mg	2	0	0	Co	2	0	0	Ag	1	0	0	
N	3	0	10	Si	4	0	0	Cu	2	0	0	I	3	0	0	
O	2	0	30	P	3	0	0	Se	2	0	0					

Error Margin (ppm):	5	DBE Range:	-2.0 - 100.0	Electron Ions:	both
HC Ratio:	unlimited	Apply N Rule:	yes	Use MSn Info:	yes
Max Isotopes:	all	Isotope RI (%):	1.00	Isotope Res:	10000
MSn Iso RI (%):	75.00	MSn Logic Mode:	OR	Max Results:	10

Event#: 1 MS(E+) Ret. Time : 0.493 -> 0.560 Scan# : 75 -> 85

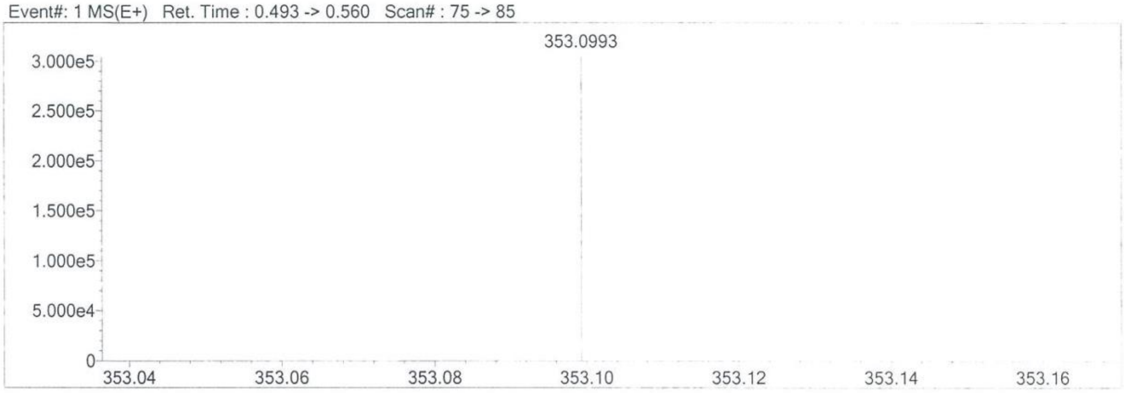

Measured region for 353.0993 m/z

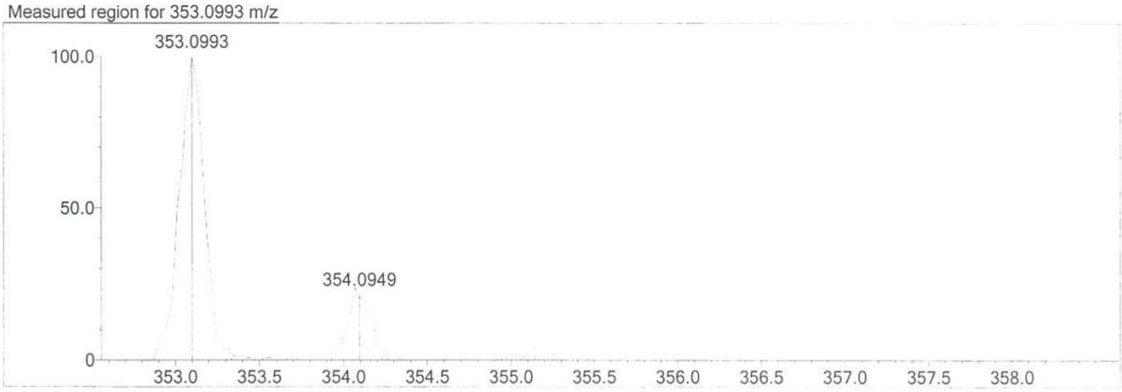

C18 H18 O6 [M+Na]+ : Predicted region for 353.0996 m/z

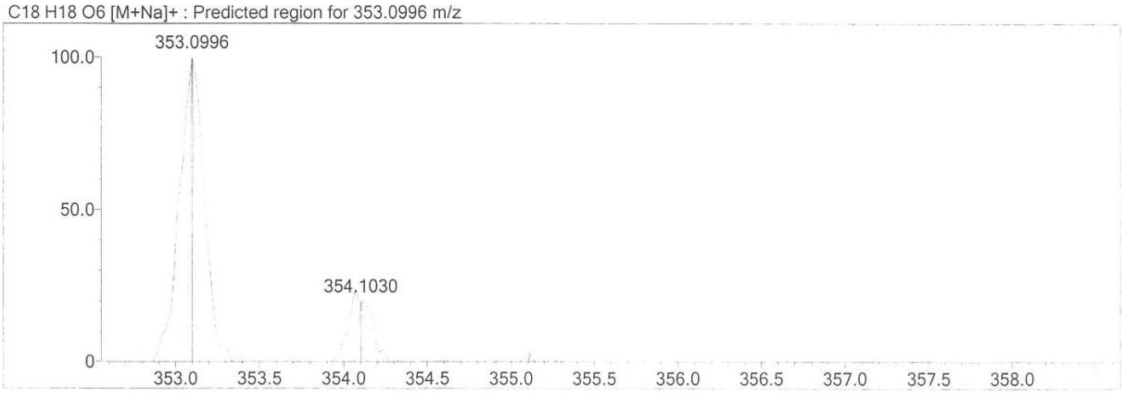

Formula (M)	Ion	Meas. m/z	Pred. m/z	Df. (mDa)	Df. (ppm)	DBE
C18 H18 O6	[M+Na]+	353.0993	353.0996	-0.3	-0.85	10.0

Figure S16 HRESIMS spectrum of 5/6.

6. NMR and MS spectra of compound 7.

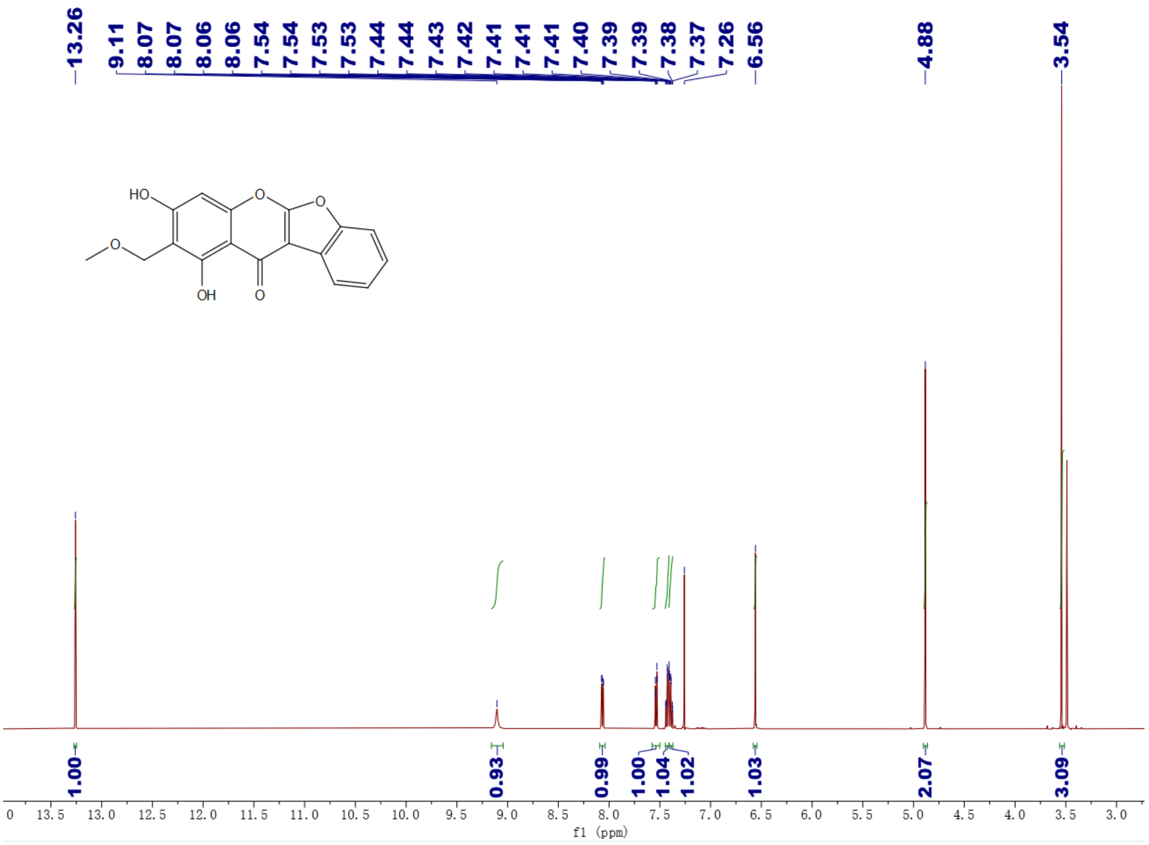

Figure S17 ^{1}H-NMR spectrum (500 MHz) of 7 in $CDCl_3$.

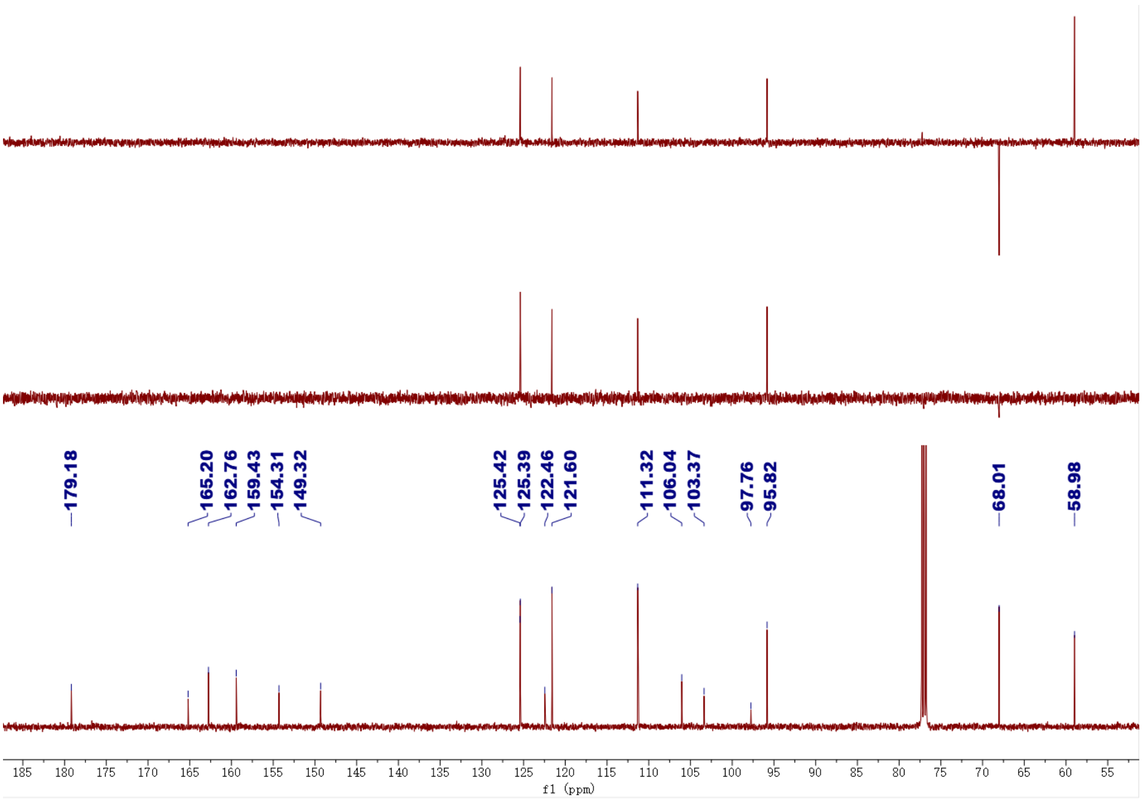

Figure S18 ^{13}C-NMR spectrum (125 MHz) of 7 in $CDCl_3$.

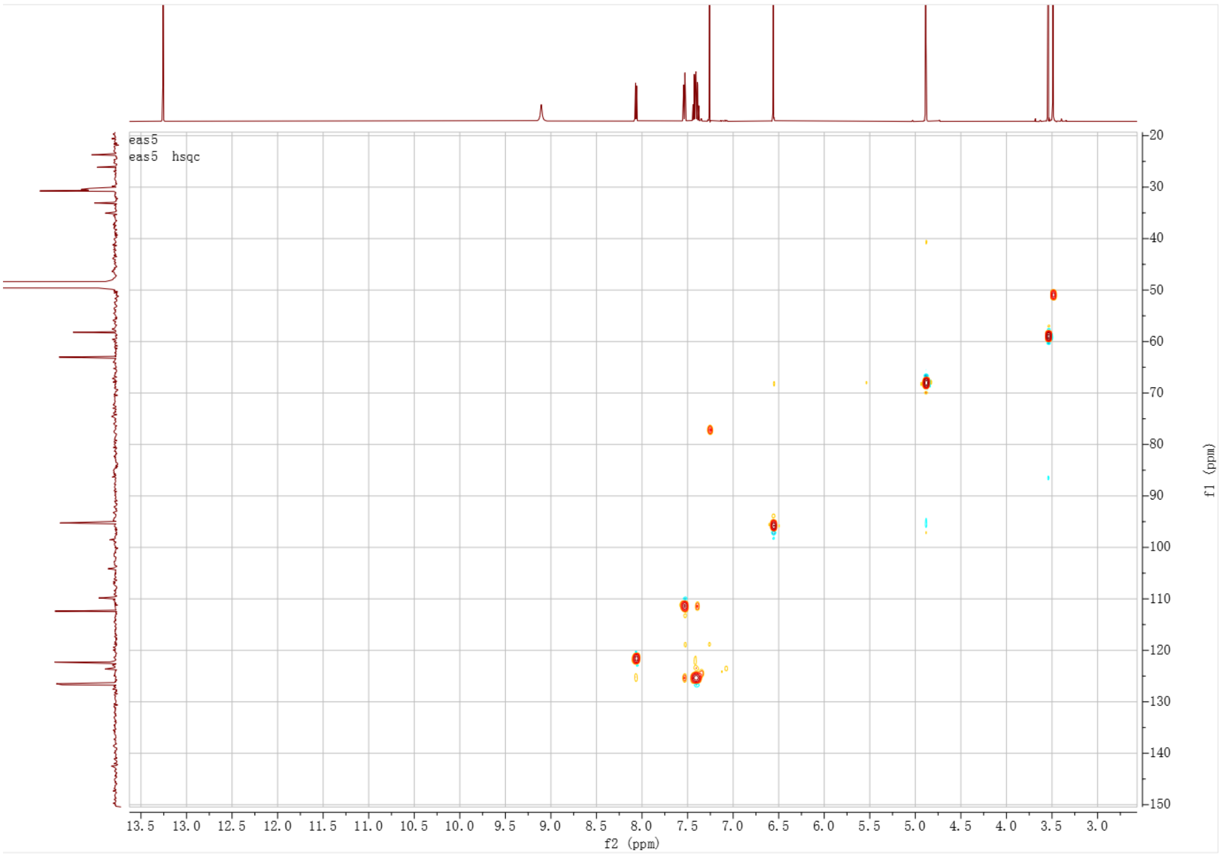

Figure S19 HSQC spectrum (500 MHz) of 7 in CDCl₃.

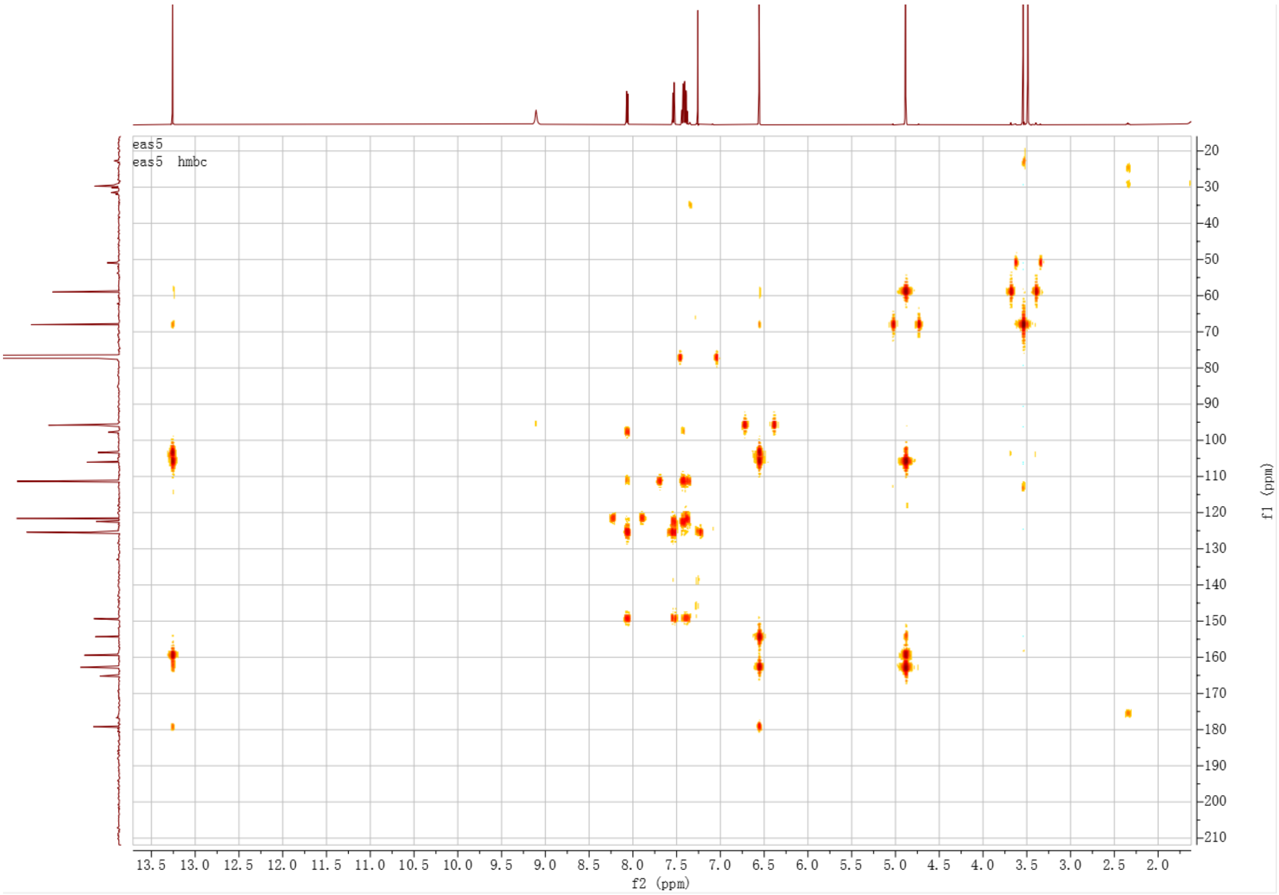

Figure S20 HMBC spectrum (500 MHz) of 7 in CDCl₃.

Data File: E:\DATA\2022\0114\EAs5.lcd

Elmt	Val.	Min	Max	Elmt	Val.	Min	Max	Elmt	Val.	Min	Max	Elmt	Val.	Min	Max	Use Adduct
H	1	5	100	F	1	0	0	Cl	1	0	0	Ag	1	0	0	H
2H	1	0	0	Na	1	0	0	Co	2	0	0	I	3	0	0	
B	3	0	0	Mg	2	0	0	Cu	2	0	0	Ir	3	0	0	
C	4	10	65	Si	4	0	0	Se	2	0	0					
N	3	0	10	P	3	0	0	Br	1	0	0					
O	2	0	30	S	2	0	5	Pd	2	0	0					

Error Margin (ppm):	5	DBE Range:	not fixed	Electron Ions:	both
HC Ratio:	unlimited	Apply N Rule:	yes	Use MSn Info:	yes
Max Isotopes:	all	Isotope RI (%):	1.00	Isotope Res:	10000
MSn Iso RI (%):	75.00	MSn Logic Mode:	OR	Max Results:	20

Event#: 2 MS(E-) Ret. Time : 0.333 -> 0.773 - 0.000 -> 0.367 Scan# : 52 -> 118 - 2 -> 58

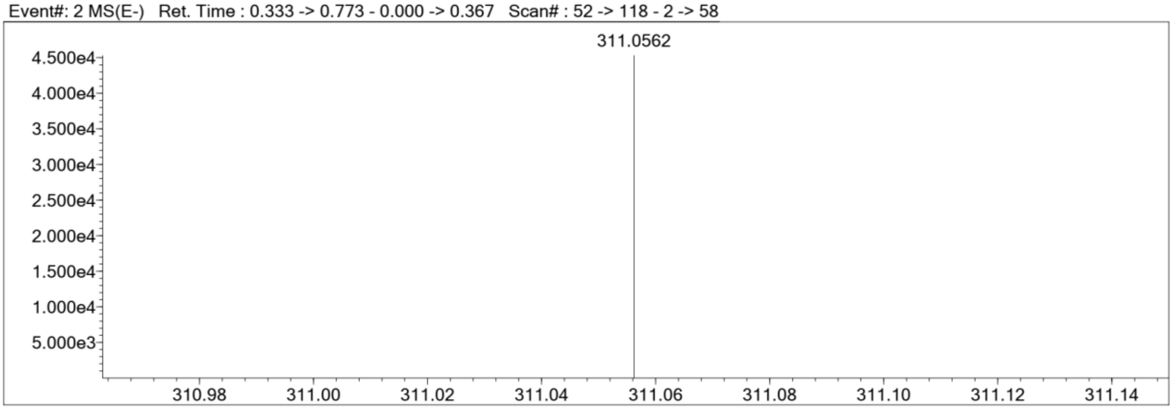

Measured region for 311.0562 m/z

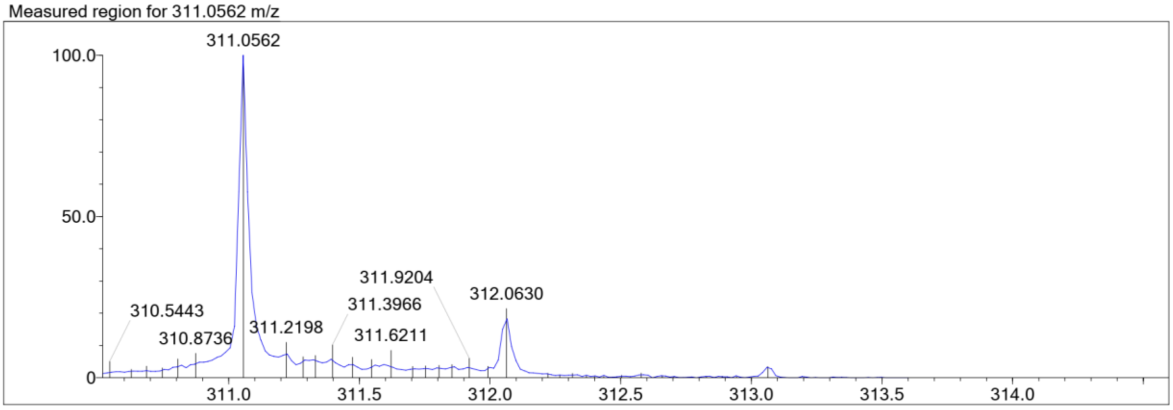

C17 H12 O6 [M-H]- : Predicted region for 311.0561 m/z

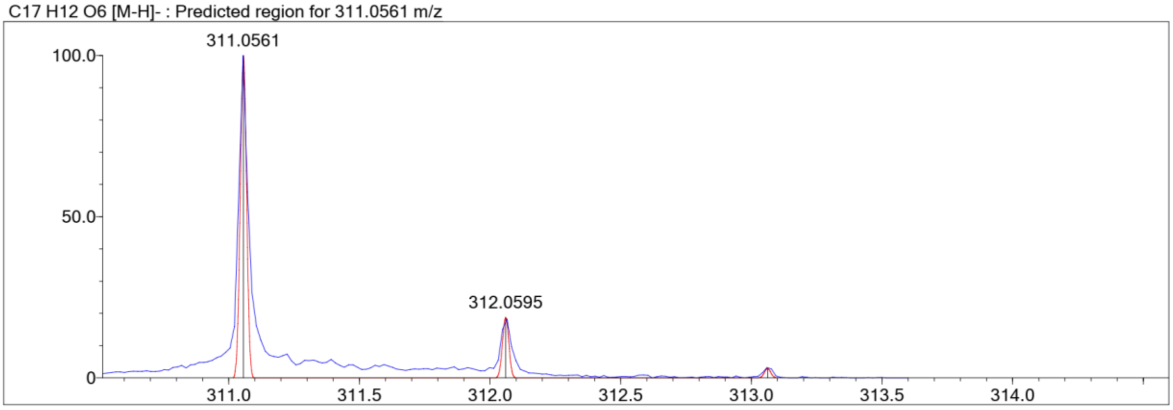

Formula (M)	Ion	Meas. m/z	Pred. m/z	Df. (mDa)	Df. (ppm)	DBE
C17 H12 O6	[M-H]-	311.0562	311.0561	0.1	0.32	12.0

Figure S21 HRESIMS spectrum of 7.

7. NMR and MS spectra of compound 8.

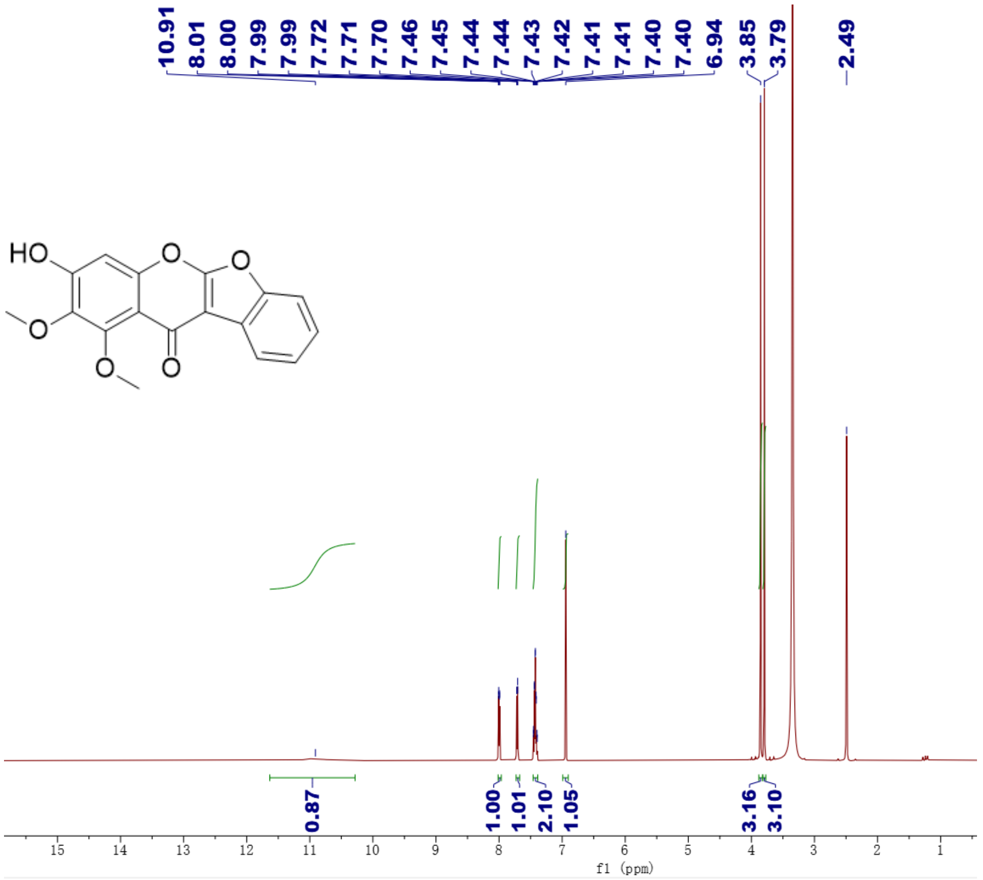

Figure S22 ^{1}H-NMR spectrum (500 MHz) of 8 in DMSO-d_6.

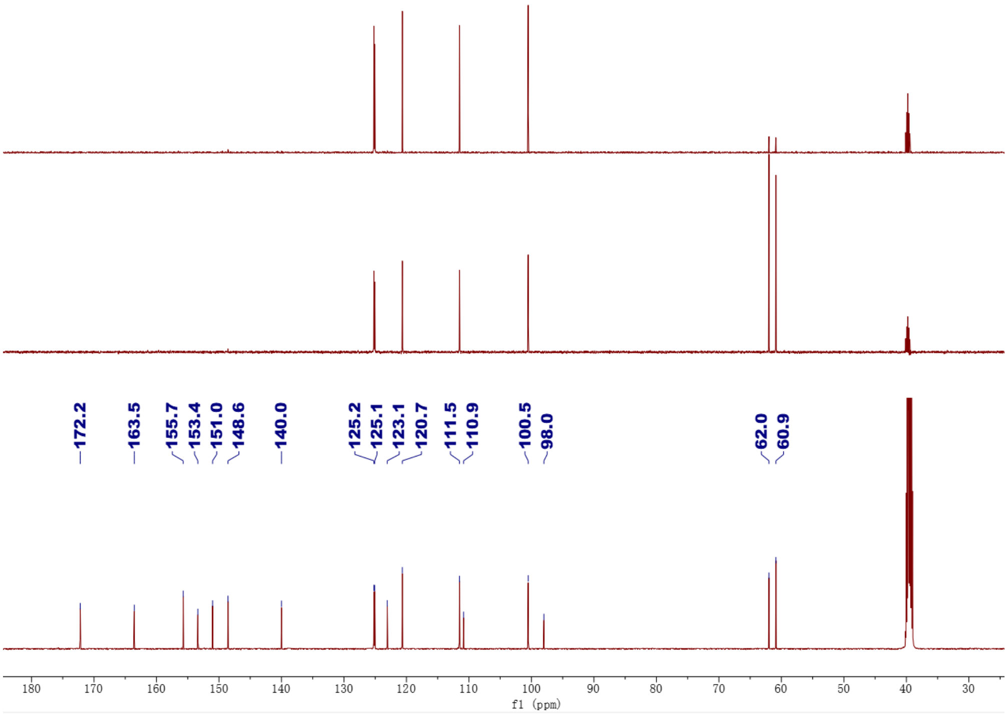

Figure S23 ^{13}C-NMR spectrum (125 MHz) of 8 in DMSO-d_6.

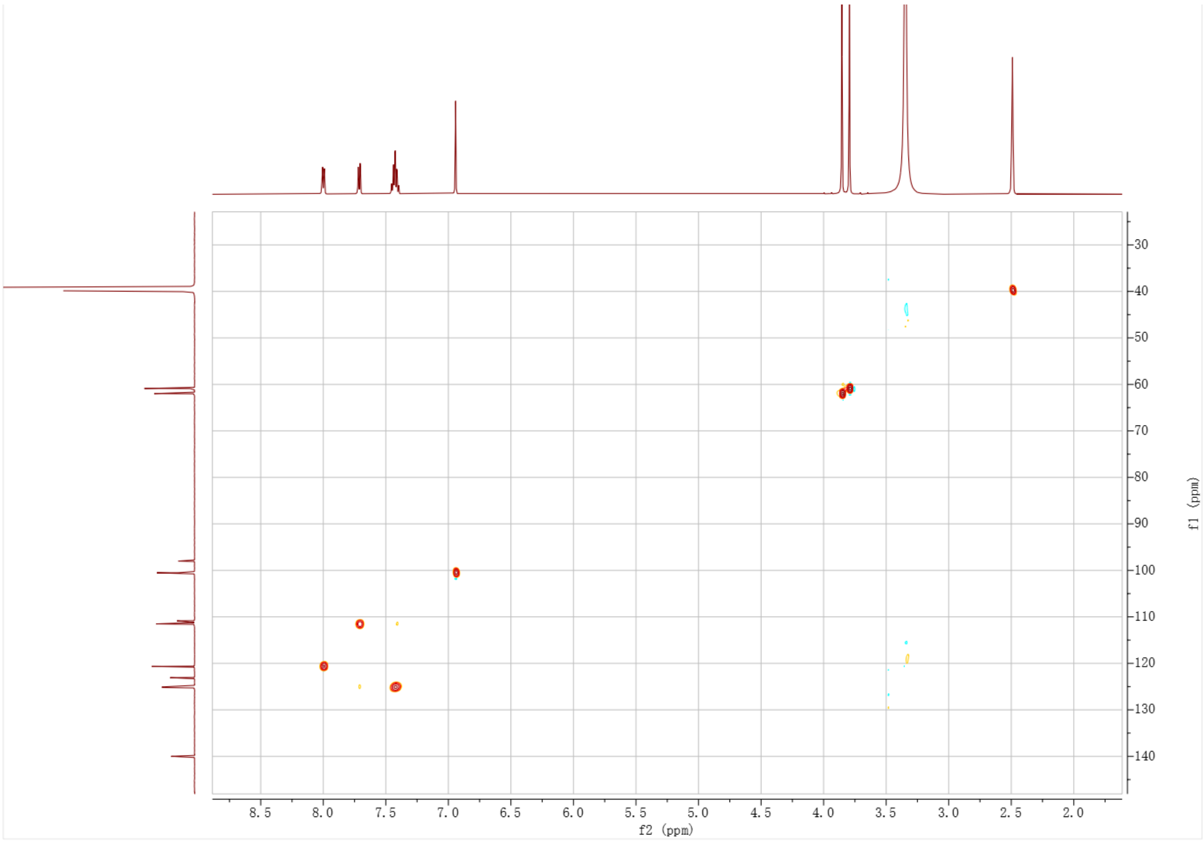

Figure S24 HSQC spectrum (500 MHz) of 8 in DMSO-*d*$_6$.

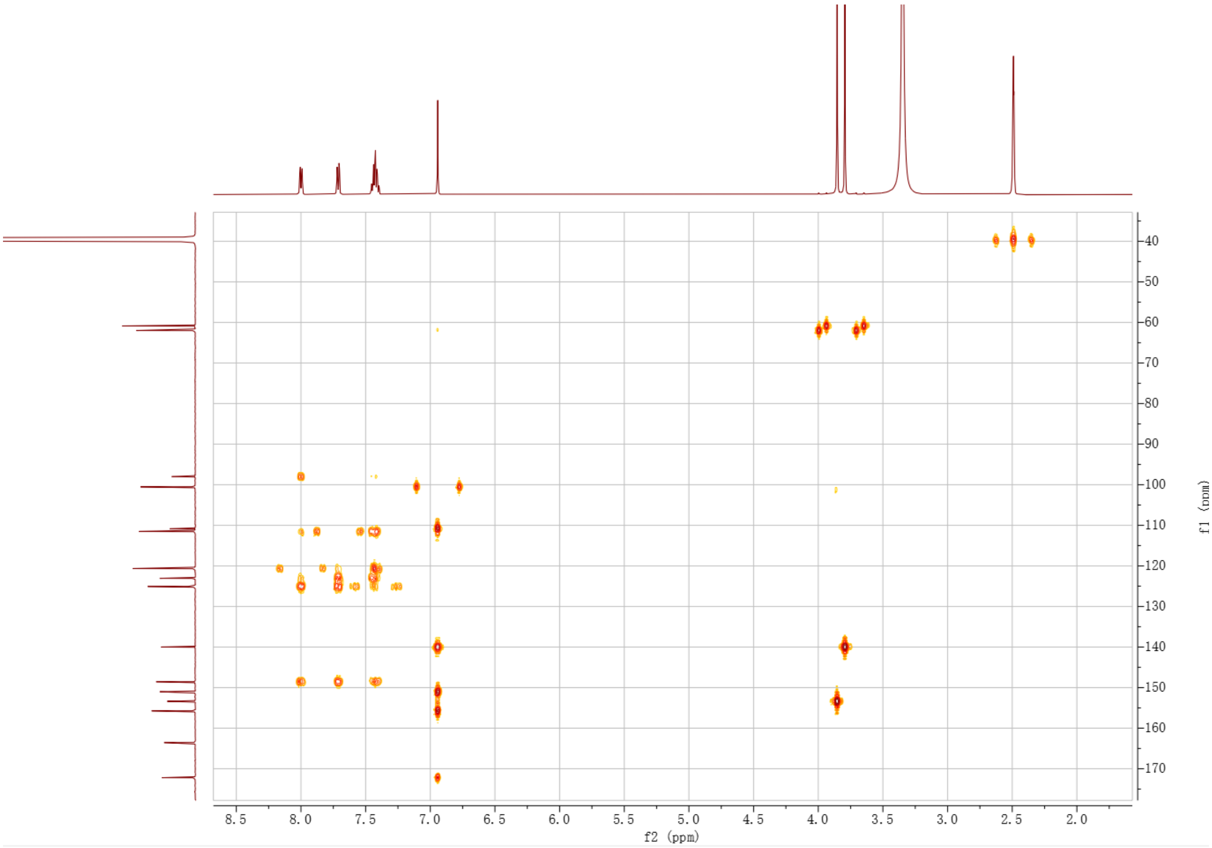

Figure S25 HMBC spectrum (500 MHz) of 8 in DMSO-*d*$_6$.

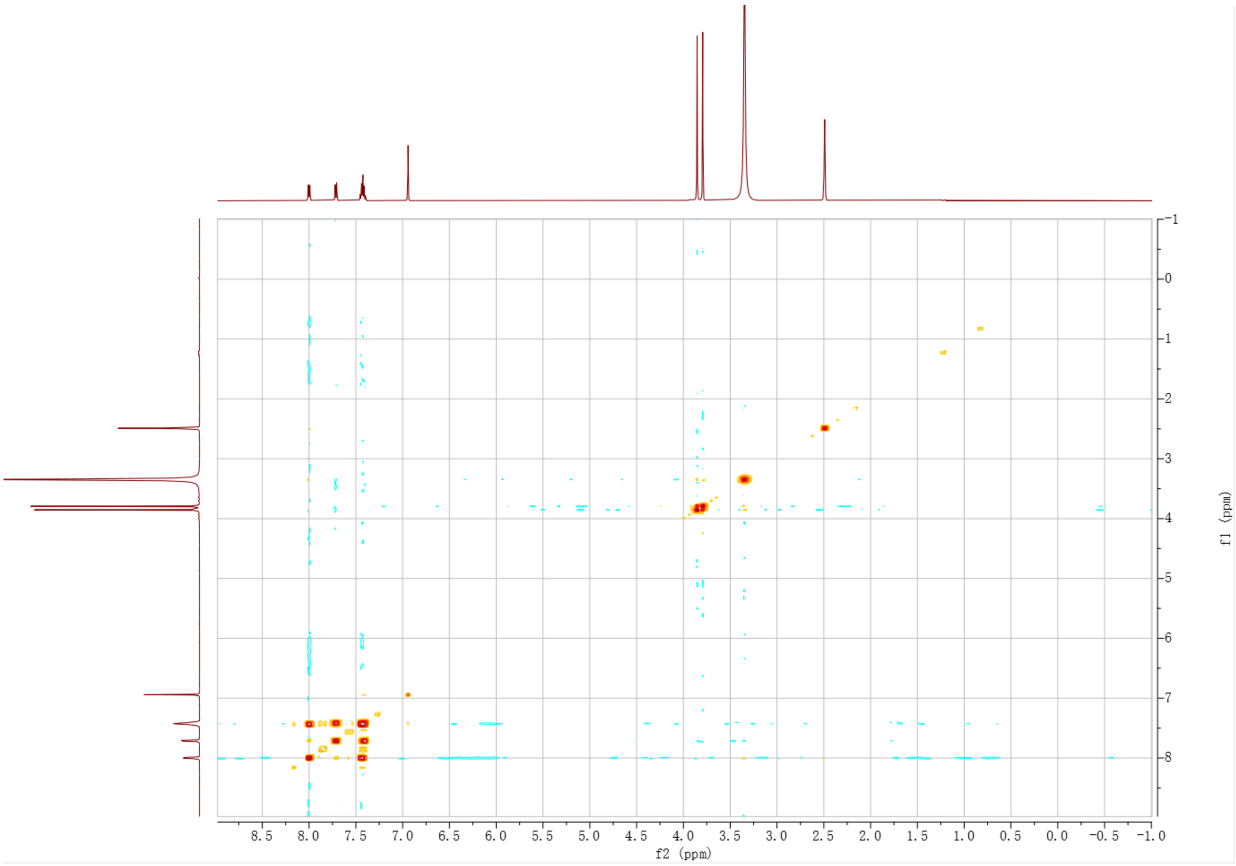

Figure S26 ^{1}H-^{1}H COSY spectrum (500 MHz) of 8 in DMSO-d_6.

Data File: E:\DATA\2021\0318\Eas49.lcd

Elmt	Val.	Min	Max	Elmt	Val.	Min	Max	Elmt	Val.	Min	Max	Elmt	Val.	Min	Max	Use Adduct
H	1	10	150	O	2	0	30	P	3	0	0	Se	2	0	0	H
2H	1	0	0	F	1	0	0	S	2	0	0	Br	1	0	0	Na
B	3	0	0	Na	1	0	0	Cl	1	0	0	Pd	2	0	0	
C	4	10	150	Mg	2	0	0	Co	2	0	0	Ag	1	0	0	
N	3	0	10	Si	4	0	0	Cu	2	0	0	I	3	0	0	

Error Margin (ppm):	5	DBE Range:	not fixed	Electron Ions:	both
HC Ratio:	unlimited	Apply N Rule:	yes	Use MSn Info:	yes
Max Isotopes:	all	Isotope RI (%):	1.00	Isotope Res:	10000
MSn Iso RI (%):	75.00	MSn Logic Mode:	OR	Max Results:	20

Event#: 1 MS(E+) Ret. Time : 0.440 -> 0.547 Scan# : 67 -> 83

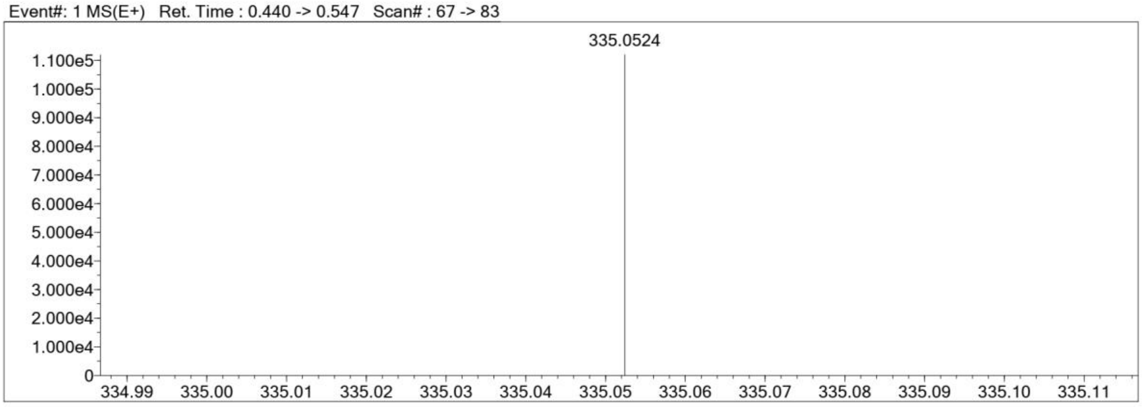

Measured region for 335.0524 m/z

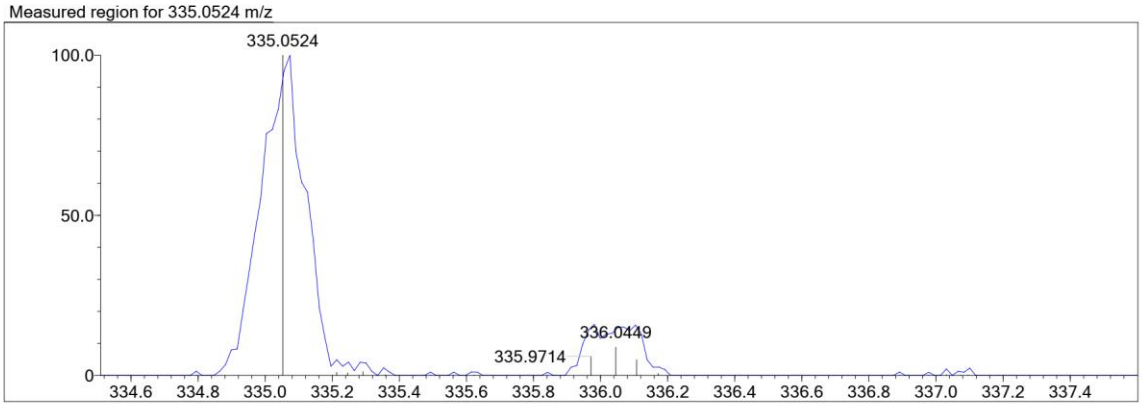

C17 H12 O6 [M+Na]+ : Predicted region for 335.0526 m/z

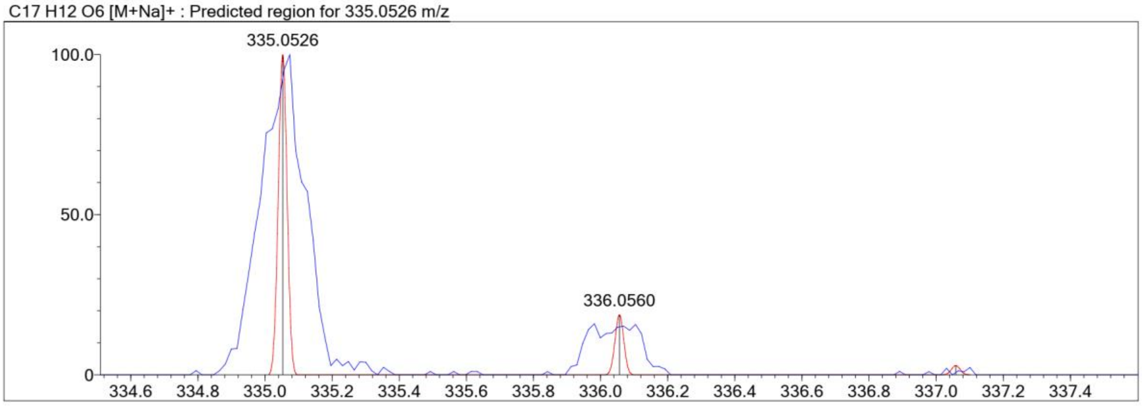

Formula (M)	Ion	Meas. m/z	Pred. m/z	Df. (mDa)	Df. (ppm)	DBE
C17 H12 O6	[M+Na]+	335.0524	335.0526	-0.2	-0.60	12.0

Figure S27 HRESIMS spectrum of 8.

8. NMR and MS spectra of compound 9.

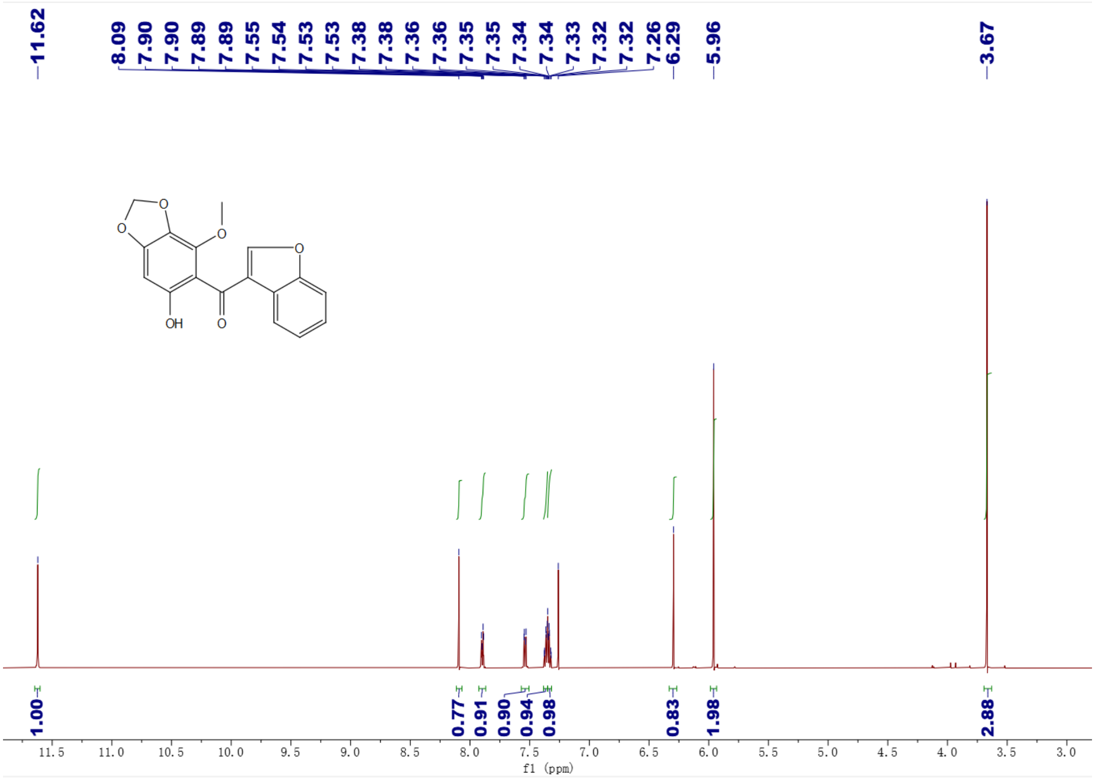

Figure S28 ^{1}H-NMR spectrum (500 MHz) of 9 in $CDCl_3$.

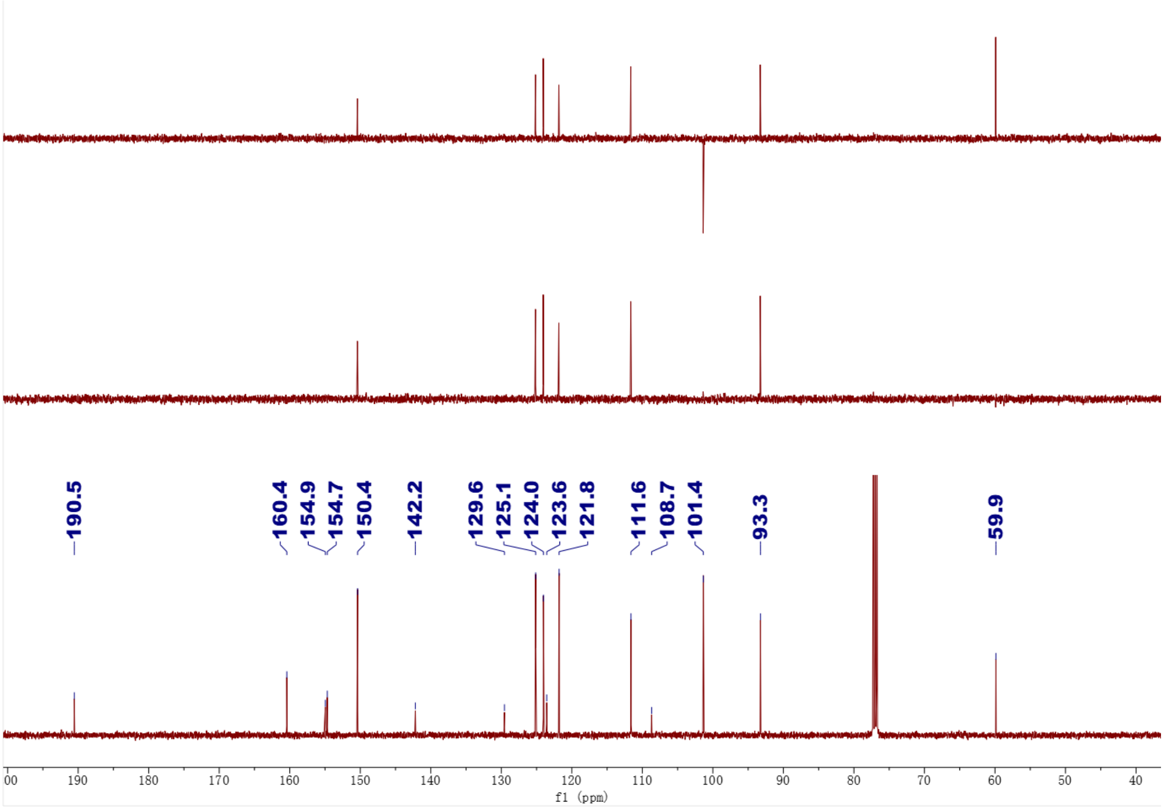

Figure S29 ^{13}C-NMR spectrum (125 MHz) of 9 in $CDCl_3$.

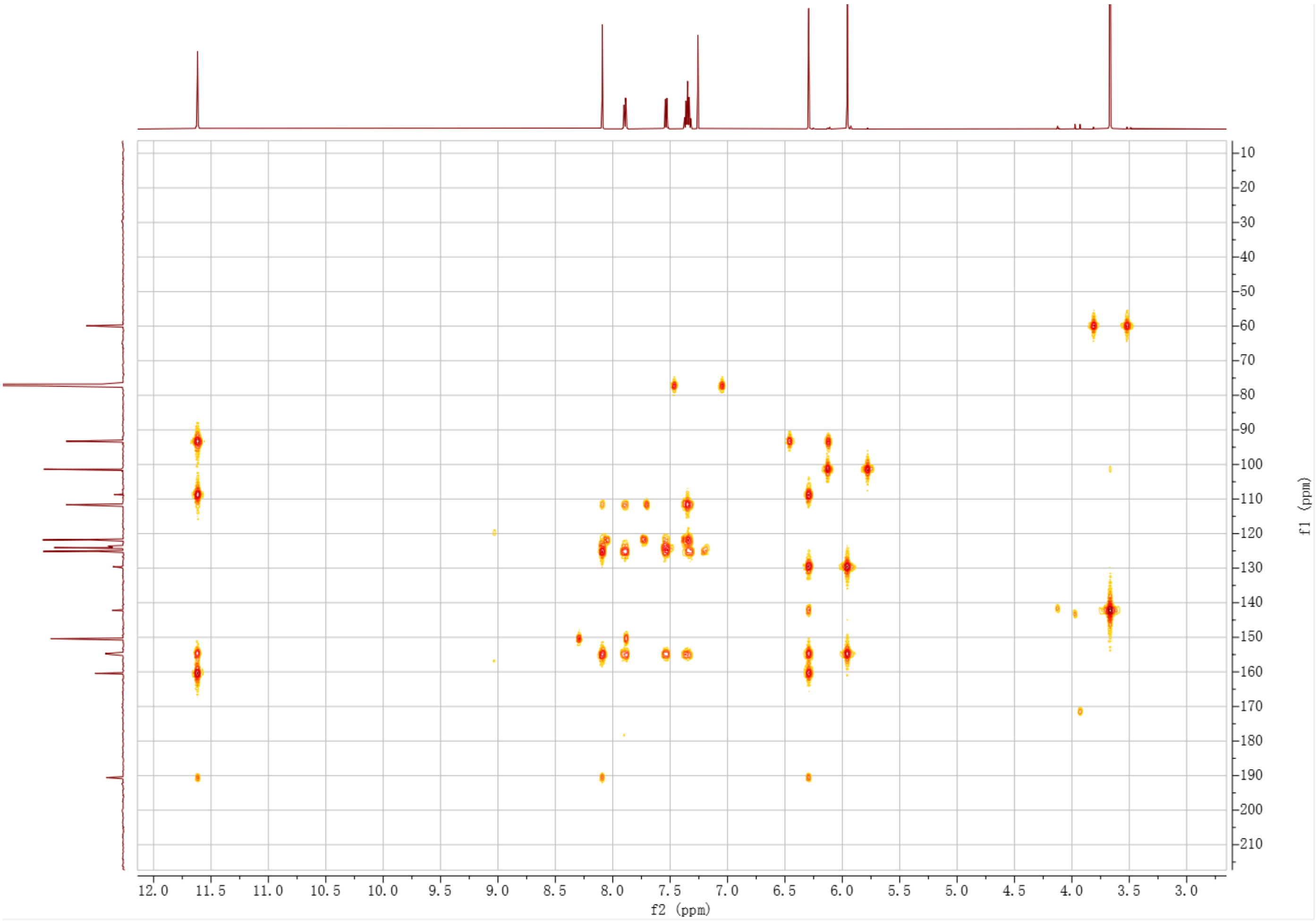

Figure S30 HMBC spectrum (500 MHz) of 9 in $CDCl_3$.

Data File: E:\DATA\2020\1209\eas7.lcd

Elmt	Val.	Min	Max	Elmt	Val.	Min	Max	Elmt	Val.	Min	Max	Elmt	Val.	Min	Max	Use Adduct
H	1	5	100	F	1	0	0	S	2	0	5	Br	1	0	0	Na
2H	1	0	0	Na	1	0	0	Cl	1	0	0	Pd	2	0	0	
C	4	5	50	Mg	2	0	0	Co	2	0	0	Ag	1	0	0	
N	3	0	10	Si	4	0	0	Cu	2	0	0	I	3	0	0	
O	2	0	30	P	3	0	0	Se	2	0	0					

Error Margin (ppm): 5
HC Ratio: unlimited
Max Isotopes: all
MSn Iso RI (%): 75.00

DBE Range: -2.0 - 100.0
Apply N Rule: yes
Isotope RI (%): 1.00
MSn Logic Mode: OR

Electron Ions: both
Use MSn Info: yes
Isotope Res: 10000
Max Results: 10

Event#: 1 MS(E+) Ret. Time : 0.400 -> 0.613 Scan# : 61 -> 93

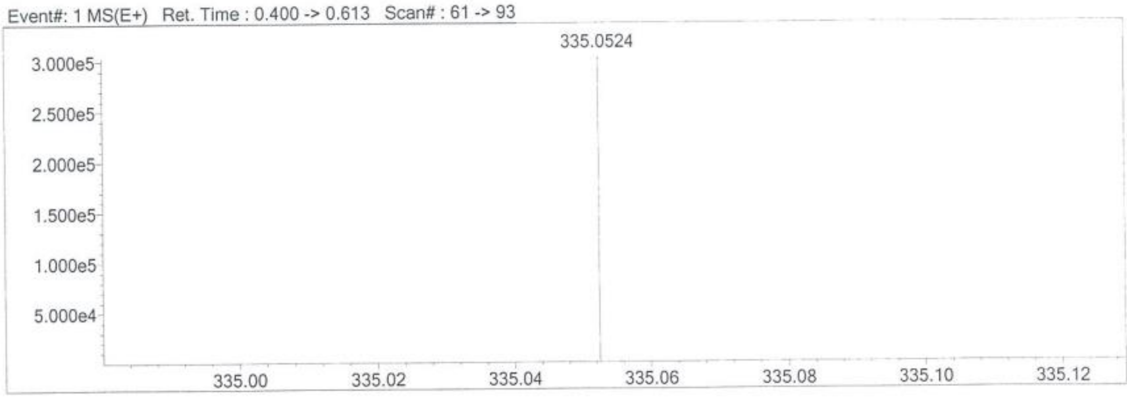

Measured region for 335.0524 m/z

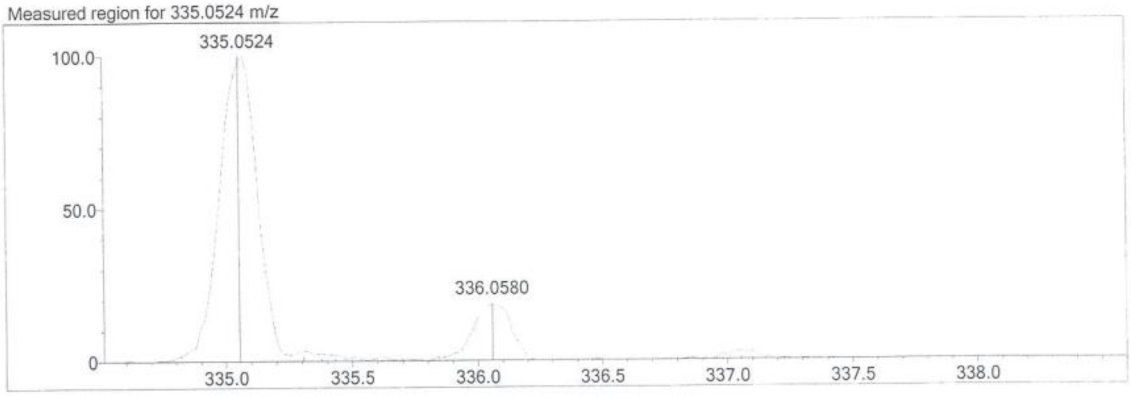

C17 H12 O6 [M+Na]+ : Predicted region for 335.0526 m/z

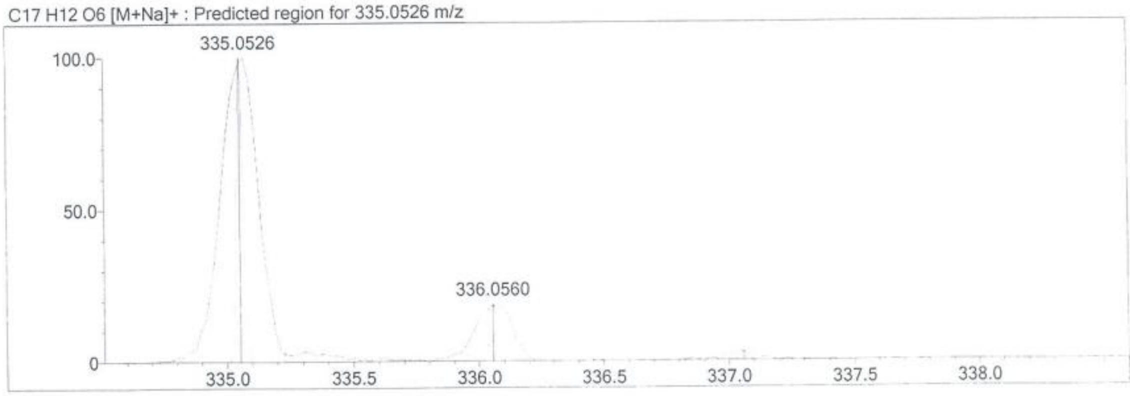

Formula (M)	Ion	Meas. m/z	Pred. m/z	Df. (mDa)	Df. (ppm)	DBE
C17 H12 O6	[M+Na]+	335.0524	335.0526	-0.2	-0.60	12.0

Figure S31 HRESIMS spectrum of 9.

9. NMR and MS spectra of compound 10.

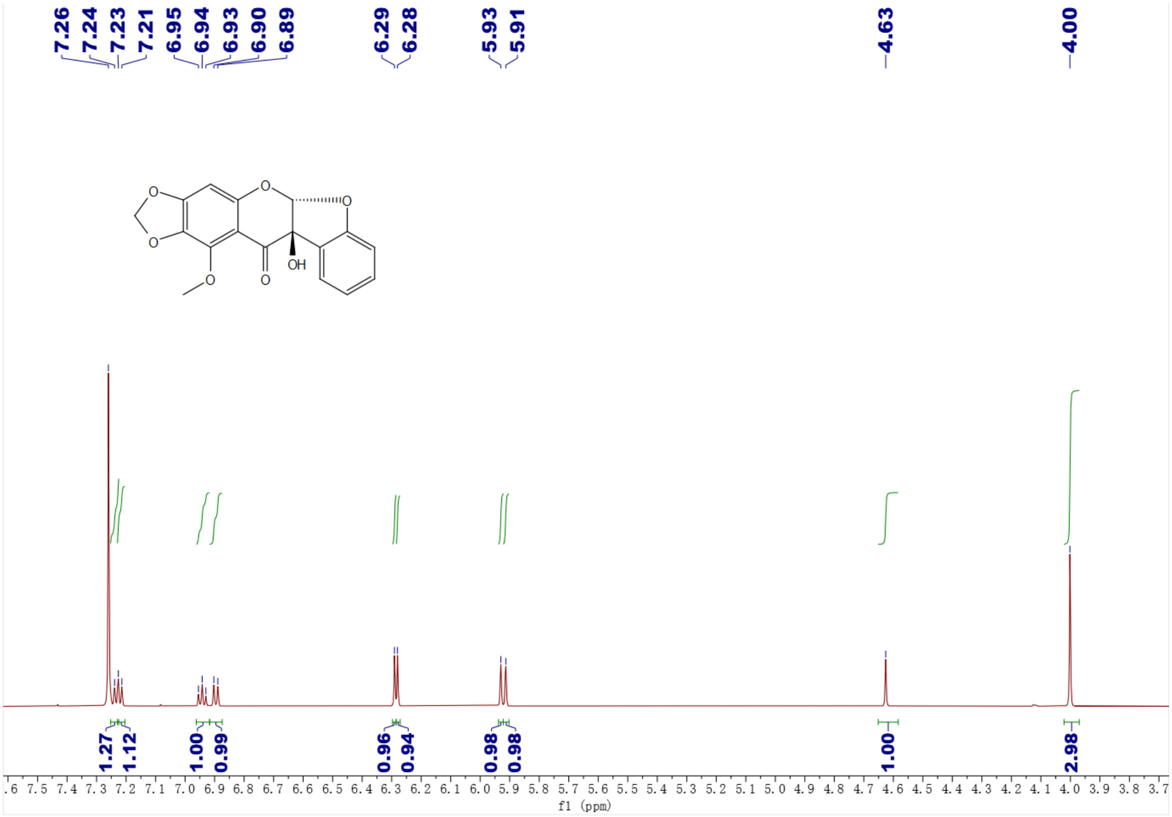

Figure S32 ^{1}H-NMR spectrum (500 MHz) of 10 in $CDCl_3$.

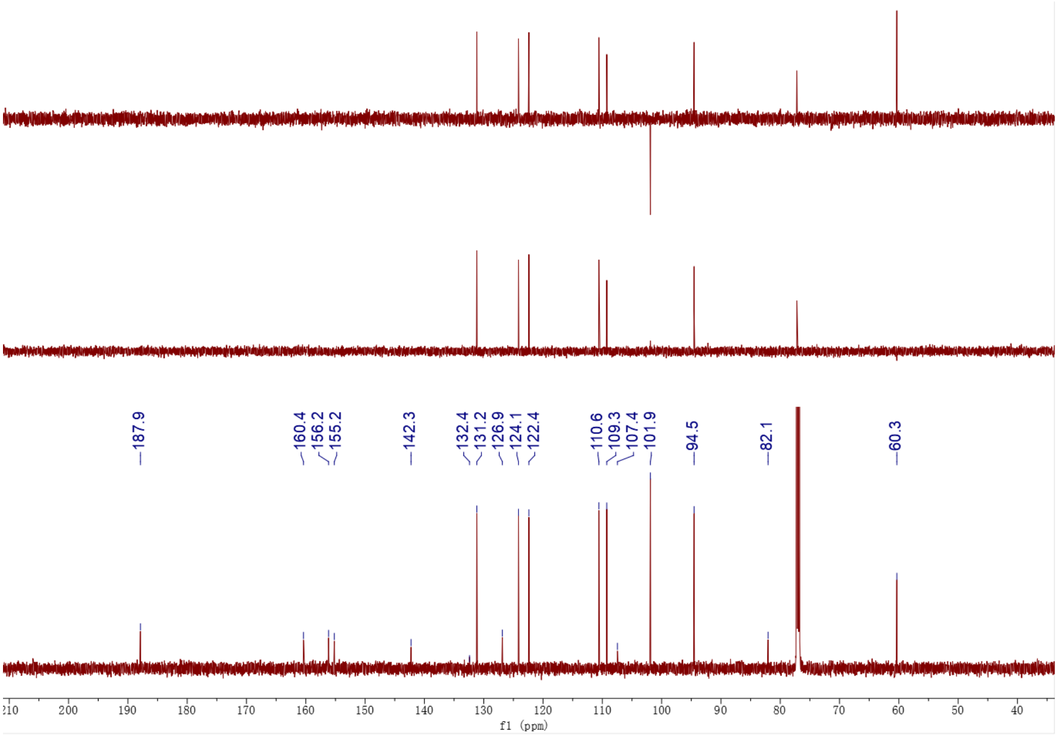

Figure S33 ^{13}C-NMR spectrum (125 MHz) of 10 in $CDCl_3$.

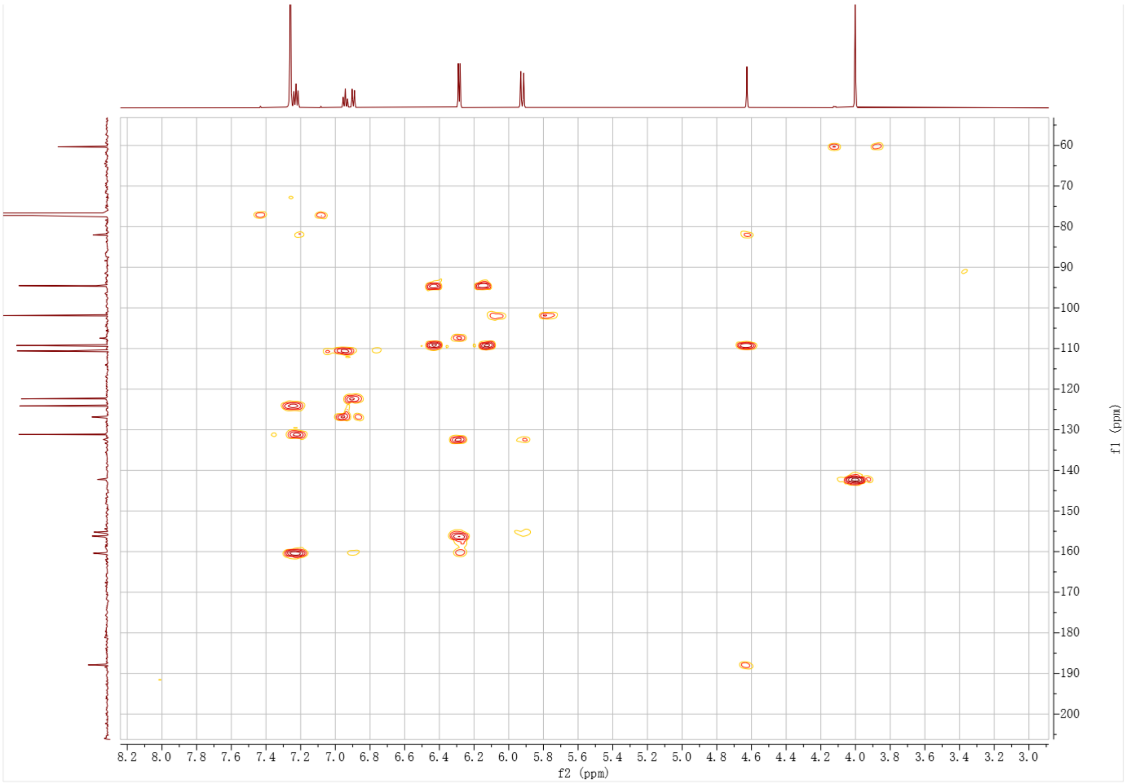

Figure S34 HMBC spectrum (500 MHz) of 10 in $CDCl_3$.

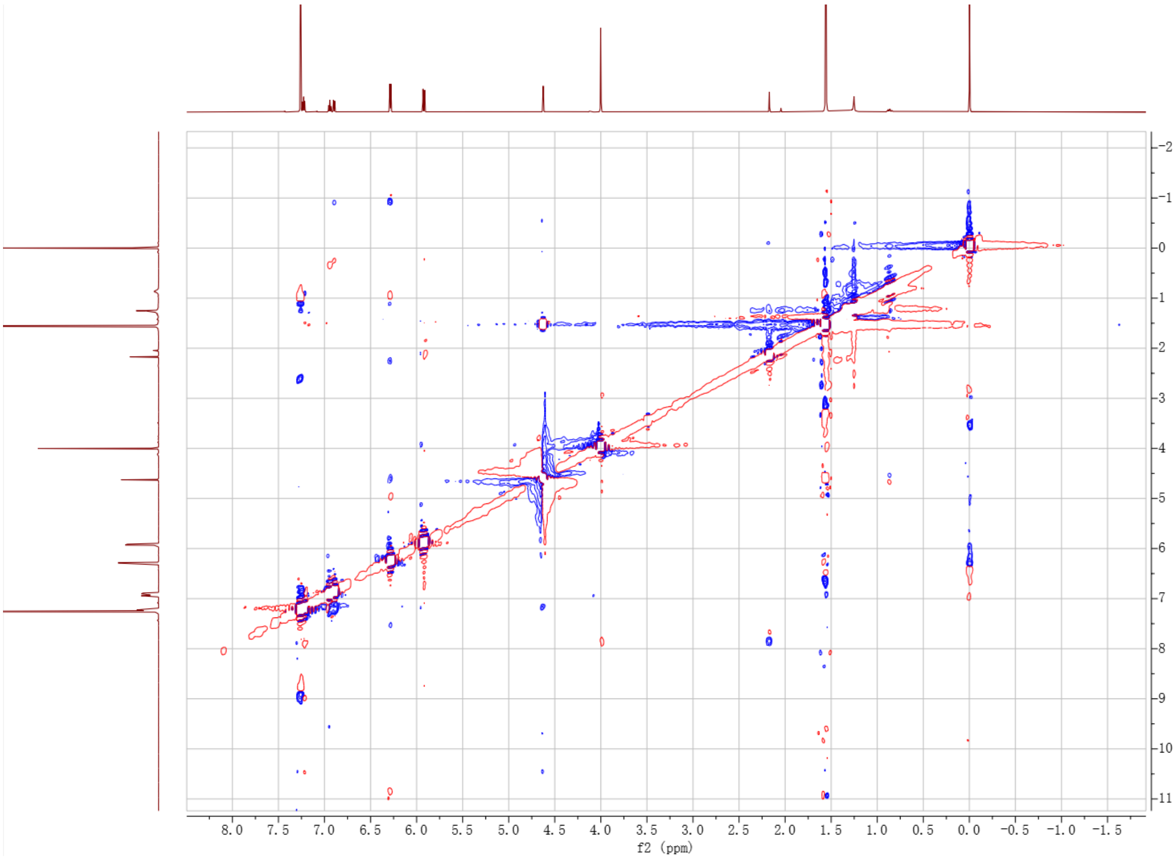

Figure S35 ROESY spectrum (500 MHz) of 10 in $CDCl_3$.

Formula Predictor Report - Eas8.lcd | Page 1 of 1

Data File: E:\DATA\2021\0414\Eas8.lcd

Elmt	Val.	Min	Max	Elmt	Val.	Min	Max	Elmt	Val.	Min	Max	Elmt	Val.	Min	Max	Use Adduct
H	1	10	100	F	1	0	0	S	2	0	0	Br	1	0	5	H
2H	1	0	0	Na	1	0	0	Cl	1	0	0	Pd	2	0	0	Na
C	4	5	50	Mg	2	0	0	Co	2	0	0	Ag	1	0	0	
N	3	0	20	Si	4	0	0	Cu	2	0	0	I	3	0	0	
O	2	0	30	P	3	0	0	Se	2	0	0					

Error Margin (ppm):	5	DBE Range:	-2.0 - 100.0	Electron Ions:	both
HC Ratio:	unlimited	Apply N Rule:	no	Use MSn Info:	yes
Max Isotopes:	all	Isotope RI (%):	1.00	Isotope Res:	10000
MSn Iso RI (%):	75.00	MSn Logic Mode:	OR	Max Results:	20

Event#: 1 MS(E+) Ret. Time : 0.360 -> 0.520 Scan# : 55 -> 79

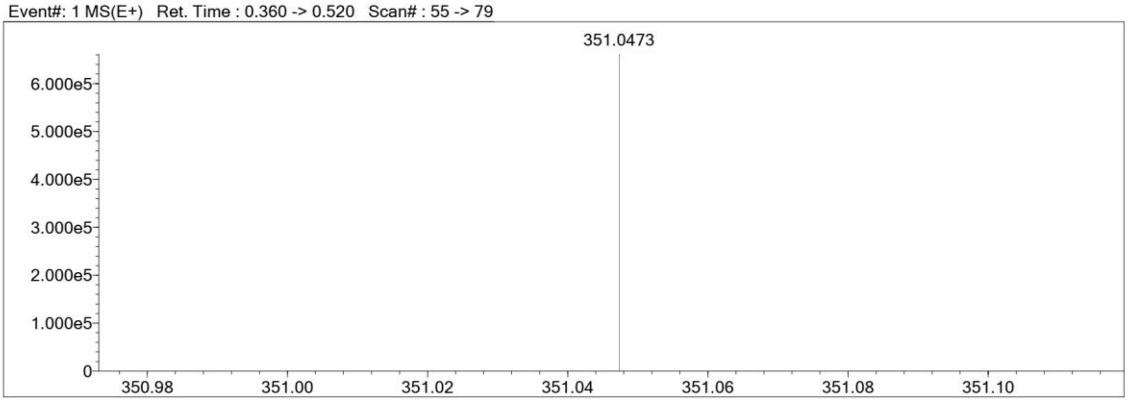

Measured region for 351.0473 m/z

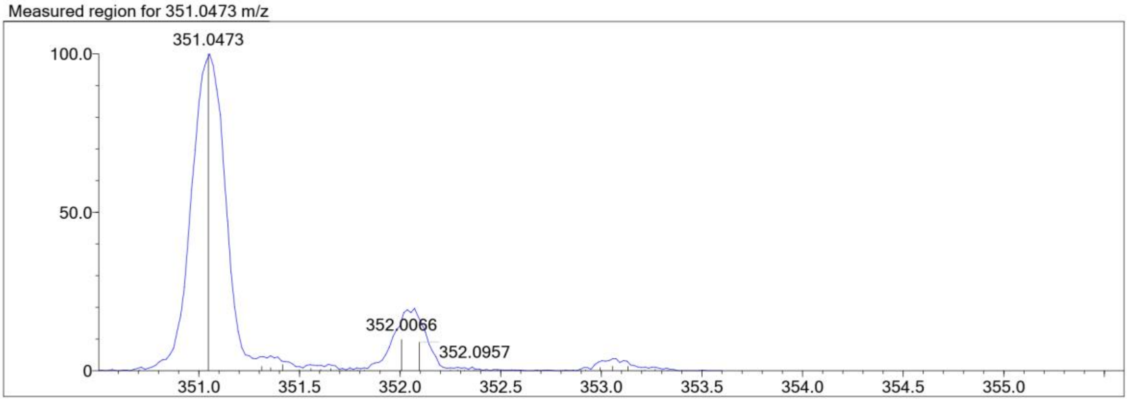

C17 H12 O7 [M+Na]+ : Predicted region for 351.0475 m/z

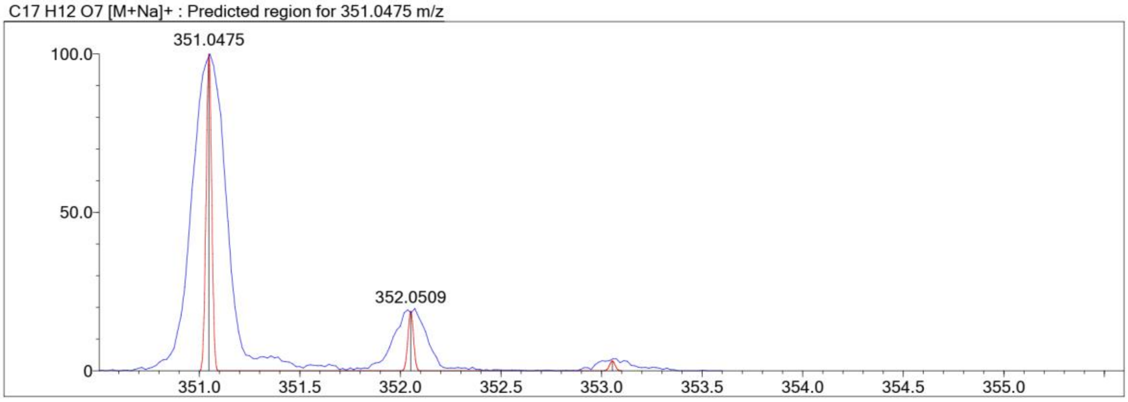

Formula (M)	Ion	Meas. m/z	Pred. m/z	Df. (mDa)	Df. (ppm)	DBE
C17 H12 O7	[M+Na]+	351.0473	351.0475	-0.2	-0.57	12.0

Figure S36 HRESIMS spectrum of 10.

References

1. Gao, Y.; Yang, J.; Yang, X. L.; Zhang, L.; Wang, J.; Li, Q.; Lin, D. M.; Zhang, M.; Xia, S. N.; Xu, L. L.; Zhang, Q.; Hai, P.; Liu Y. H.; Wang, S.; Guo, L. P. Novel dibenzofuran and biphenyl phytoalexins from *Sorbus pohuashanensis* suspension cell and their antimicrobial activities. *Fitoterapia* **2021**, *152*, 104914..
2. Frisch, M.J., Trucks, G.W.; Schlegel, H.B.; Scuseria G.E.; Robb M. A.; Cheeseman J. R.; Scalmani G.; Barone V.; Petersson G. A.; Nakatsuji H.; Li X.; Caricato M.; Marenich A.; Bloino J.; Janesko B. G.; Gomperts R.; Mennucci B.; Hratchian H. P.; Ortiz J. V.; Izmaylov A. F.; Sonnenberg J. L.; Williams-Young D.; Ding F.; Lipparini F.; Egidi F.; Goings J.; Peng B.; Petrone A.; Henderson T.; Ranasinghe D.; Zakrzewski V. G.; Gao J.; Rega N.; Zheng G.; Liang W.; Hada M.; Ehara M.; Toyota K.; Fukuda R.; Hasegawa J.; Ishida M.; Nakajima T.; Honda Y.; Kitao O.; Nakai H.; Vreven T.; Throssell K.; Montgomery J. A. jr.; Peralta J. E.; Ogliaro F.; Bearpark M.; Heyd J. J.; Brothers E.; Kudin K. N.; Staroverov V. N.; Keith T.; Kobayashi R.; Normand J.; Raghavachari K.; Rendell A.; Burant J. C.; Iyengar S. S.; Tomasi J.; Cossi M.; Millam J. M.; Klene M.; Adamo C.; Cammi R.; Ochterski J. W.; Martin R. L.; Morokuma K.; Farkas O.; Foresman J. B.;, Fox D. J. Gaussian 16, Revision A.03; Gaussian Inc.: Wallingford, **2016**.
3. Hai, P.; Jia, H.; Luo, Z.; Fan, H.; He, Y.; Li, X. Meroterpenoids with anti-triple negative breast cancer and antimicrobial activities from *Arnebia euchroma* Fitoterapia, **2024**, *179*, 106234.
4. Bruhn, T.; Schaumlöffel, A.; Hemberger, Y.; Pecitelli, G. SpecDis, 1.71: Berlin, Germany, **2017**.
